# MicroRNA-33b downregulates the differentiation and development of porcine preadipocytes

**DOI:** 10.1007/s11033-013-2954-z

**Published:** 2014-01-08

**Authors:** Masaaki Taniguchi, Ikuyo Nakajima, Koichi Chikuni, Misaki Kojima, Takashi Awata, Satoshi Mikawa

**Affiliations:** 1 Animal Genome Research Unit, Agrogenomics Research Center, National Institute of Agrobiological Sciences, 2-1-2 Kannondai, Tsukuba, Ibaraki 305-8602 Japan; 2 Animal Products Research Division, National Institute of Livestock and Grassland Science, 2 Ikenodai, Tsukuba, Ibaraki 305-0901 Japan

**Keywords:** Adipocyte, Gene expression, Lipogenesis, MicroRNA, PPAR, SREBF

## Abstract

Sterol regulatory element binding transcription factor (SREBF) is a key transcription regulator for lipid homeostasis. MicroRNA-33b (miR-33b) is embedded in intron 16 of porcine *SREBF1* and is conserved among most mammals. Here, we investigated the effect of miR-33b on adipocyte differentiation and development in porcine subcutaneous pre-adipocytes (PSPA). PSPA were transiently transfected with miR-33b, and adipose differentiation was then induced. Delayed adipose differentiation and decreased lipid accumulation were observed in miR-33b-transfected PSPA. Computational predictions suggested that miR-33b may target early B cell factor 1 (EBF1), an adipocyte activator of lipogenesis regulators such as CCAAT-enhancer binding protein alpha (C/EBPα) and peroxisome proliferator-activated receptor gamma (PPARγ). Both gene and protein expression of EBF1 were downregulated in miR-33b-transfected PSPA, followed by considerable decreases in the expression of *C/EBPα* and *PPARγ* and their downstream lipogenic genes. However, miR-33b transfection did not markedly affect mRNA and protein expression of SREBF1. We also investigated differences in the expression of miR-33b and lipogenic genes in subcutaneous fat tissues between 5-month-old crossbred gilts derived from Landrace (lean-type) and Meishan (fatty-type) boars. Landrace-derived crossbred gilts expressed more miR-33b and less lipogenic genes than did gilts derived from Meishan. Our results suggest that miR-33b affected the differentiation and development of PSPA by attenuating the lipogenic gene expression cascade through EBF1 to C/EBPα and PPARγ. The differential expression of miR-33b observed in crossbred gilts may in part account for differences in lipogenic gene expression and the fat:lean ratio between pig breeds.

**Electronic supplementary material:**

The online version of this article (doi:10.1007/s11033-013-2954-z) contains supplementary material, which is available to authorized users.

## Introduction

Pork, together with chicken and beef, is an important protein source for humans. In the livestock industry, including pork production, carcass fat quantity and quality are major determinants of the productivity and palatability of meat. Previous work has shown that the molecular pathway of fat metabolism is regulated in a species-specific and fat-depot-specific manner [1, 2]. Therefore, it is essential to understand the molecular mechanisms that underlie adipogenesis and lipogenesis in porcine fat tissues.

Previous studies have investigated the transcriptional regulation of genes associated with adipogenesis [3, 4]. Among the transcription regulators, sterol regulatory element-binding transcription factor (SREBF) is known to regulate the transcriptional activation of genes involved in the uptake and synthesis of cholesterol, fatty acids, triglycerides, and phospholipids [5–7]. In addition to transcription factors, microRNAs (miRNA), which are ~22-nt non-coding RNAs generated from the sequential processing of long RNA transcripts [8], are considered to play key roles in the regulation of gene expression at the post-transcriptional level in the diverse regulatory pathways of many cellular processes. MirBase, a public database of miRNA, contains 331 miRNAs that have been identified in the porcine genome (Release 19 available since August 2012, http://www.mirbase.org) [9]. Recent studies [10, 11] reported that miRNA (miR-33a) embedded in *SREBF2* gene influences cholesterol metabolism in murine hepatocytes and human macrophages by repressing adenosine-triphosphate-binding cassette transporter A1 (ABCA1). In addition, Dávalos et al. [12] reported that miR-33a/b is associated with the repression of fatty acid oxidation and insulin signaling in hepatocytes. Further, identification and functional studies of miRNAs have been done in livestock species including pig and cattle [13, 14]. These studies suggest that fat cell development is achieved via a complicated mechanism that is potentially regulated by miRNAs in a species-specific manner.

Therefore, to clarify the porcine-specific pathways in adipocyte differentiation and development, we asked whether miR-33b influences the regulation of adipogenesis and lipogenesis in porcine preadipocytes. First, we determined the full-length nucleotide sequence of the porcine *SREBF1* gene. Second, we transfected miR-33b into porcine subcutaneous pre-adipocytes (PSPA) to examine whether the transcriptional regulation of genes relevant to adipocyte differentiation and development was affected by miR-33b. We also investigated the mRNA and protein expression levels of an miR-33b target gene that was identified in an miRNA target prediction search and, as such, assumed to be involved in adipocyte differentiation and development. Last, we discuss the possible effect of miR-33b on differences in backfat (BF) thickness and blood triglyceride levels between representative lean and fat crossbred pigs.

## Materials and methods

### Animal samples

European crossbred (E) dams, including Landrace, Large White, and Duroc, were mated with Landrace (L) or Meishan (M) boars. Meishan pigs are substantially fatter, particularly in subcutaneous adipose tissue, than are typical European pig breeds [15, 16]. Seven each of EL and EM gilts were fed under the same nutritional conditions until slaughter at 5 months old at the National Institute of Livestock and Grassland Science (NILGS). Body weights (mean ± SD) of the EL and EM at slaughter were 82.0 ± 3.8 and 82.8 ± 6.2, respectively. There was no significant body weight difference between the breed types (*P* = 0.782 by Student *t* test). Tissue samples for total RNA extraction were collected from the liver, longissimus dorsi (LD) muscle, and subcutaneous adipose tissues at the mid-dorsal area of these animals. BF thickness measurements at the shoulder, back, and lumber positions were averaged and are presented as such. All animal care and use in this study was in accordance with the animal experimentation guideline of the NILGS and was approved by the NILGS Animal Care Committee.

### Cell culture

PSPA were cultured in DMEM growth medium containing a low glucose concentration (1.0 g/L) and 10 % fetal bovine serum (FBS) (Invitrogen, Carlsbad, CA, USA). The subconfluent cells were passed every 3 days. To produce mature adipocytes, we plated the PSPA at 2.1 × 10^4^ cells/cm^2^ and grew them for 3 days to reach confluence. After the cells reached confluence (denoted as 0 day), adipose conversion was carried out in high-glucose (4.5 g/L) DMEM containing 10 % FBS, 5 μg/mL insulin (Sigma-Aldrich, Basel, Switzerland), 0.25 μM dexamethasone (Sigma-Aldrich), 33 μM biotin (Wako Pure Chemicals, Osaka, Japan), 17 μM pantothenate (Sigma-Aldrich), and 5 mM octanoate (Sigma-Aldrich). The medium was changed every other day. To investigate the effect of miR-33b on these cells, we subjected groups of cells to the following three experimental conditions: (1) growth medium alone; (2) differentiation with a negative control miRNA (miR-NC) transfection; and (3) differentiation with miR-33b transfection. All cells were cultured at 37 °C in a humidified incubator in 5 % CO_2_.

### Transfection

To investigate the effect of miR-33b, we transfected a commercially available mature miRNA molecule, Pre-miR™ miRNA Precursor (hsa-miR-33b) (Ambion, Austin, TX, USA), into confluent cells. The transfectant hsa-miR-33b is not a hairpin construct but a mature miRNA molecule, so that although it was designed for use with human cells, it can be applicable to pigs, cattle, or dogs, because the mature miRNA sequence of miR-33b is 100 % identical among these species. For the miR-NC in experimental group 2, we used Pre-miR™ Negative Control #1, which is a Pre-miR™ molecule designed to produce no identifiable effects on known miRNA function (Ambion). These transfectants were transfected by using the DharmaFECT 1 transfection reagent according to the manufacturer’s instructions (ThermoFisher Scientific, Waltham, MA, USA). Four replicate samples of cells were harvested at 2, 4, 8, 12, and 16 days after transfection and induction of adipocyte differentiation.

### Cloning of the porcine *SREBF1* gene

A porcine bacterial artificial chromosome (BAC) library constructed from a boar of Large White/Landrace/Duroc composite was screened by means of a PCR-based method with primers derived from the porcine *SREBF1* mRNA sequence (GenBank ID: NM_214157.1). BAC clones were sequenced to determine the full-length porcine *SREBF1* gene in accordance with the previously established method [17].

### Gene expression assay

Total RNA including small RNA was extracted from porcine tissue samples and PSPA by using ISOGEN II (NIPPON GENE, Toyama, Japan). Total RNAs were reverse-transcribed to synthesize single-strand DNA by using ReverTra Ace (TOYOBO, Osaka, Japan). Real-time PCR was performed with the TaqMan system (Applied Biosystems, Foster City, CA, USA) to examine the relative gene expression of *SREBF1*, *C/EBPa*, *EBF1*, fatty acid synthase (*FASN*), peroxisome proliferator-activated protein gamma 1 and 2 (*PPARγ1* and *PPARγ2*), stearoyl-CoA desaturase 1 (*SCD1*), adipocyte-fatty acid binding protein (*aP2*), adiponectin (*ADIPOQ*), and fatty acid translocase (*CD36*) by using primers and gene-specific probes (Table 1). The TaqMan MicroRNA Reverse Transcription Kit and TaqMan MicroRNA Assays (Applied Biosystems) designed for hsa-miR-33b were used for relative quantification of miR-33b. The TaqMan Endogenous Control Eukaryotic 18S rRNA gene (Applied Biosystems) was used for the relative quantification of all of the genes examined. The comparative threshold cycle method (ΔΔCt) was employed to calculate the relative quantification of gene expression based on the formula, 2^(−ΔΔCt)^ where

$$ \Updelta \Updelta {\text{Ct}} = (\text{Ct}_{\text{targert\;gene}} - \text{Ct}_{18S\;rRNA} )_{\text{test}} - (\text{Ct}_{\text{targert\; gene}} - \text{Ct}_{18S\;rRNA} )_{\text{calibrator}} $$

[18, 19]. The ΔΔCt values obtained from 0 day, non-treated control PSPA of experimental group 1 and from LD muscle were used as calibrators for the relative quantification of gene expression in the PSPA and 5-month-old gilts, respectively.

**Table 1:** Nucleotide sequences of primers and probes used for real-time PCR

Gene	Nucleotide sequence of primers and probes (5′–3′)	Accession number
*SREBF1*		
Forward	CGGACGGCTCACAATGC	AB686492
Reverse	GCAAGACGGCGGATTTATTC	
Probe	TCAACGACAAGATCATCGAG	
*PPARγ1*		
Forward	CTCGGACACCGGAGCTGG	AJ006756
Reverse	CAACCATGGTCACCTCGCTAA	
Probe	CGCCAGGCCACCACCGCAGATT	
*PPARγ2*		
Forward	GGTGAAACTCTGGGAGATTCTCTTA	AF059245
Reverse	CAACCATGGTCACCTCTTGTGA	
Probe	CGATGCCTTCGACACGCTGTCTGCAA	
*C/EBPα*		
Forward	AGGAGGACGAGTCGAAGCA	XM_003127015
Reverse	GGCGGAGGGTGTGAATGC	
Probe	CTTTCCCTACCAGCCACCGCCGC	
*EBF1*		
Forward	ATGTTTGTCCATAATAACTCCAAGC	XM_003359834
Reverse	CTTTGATACAGGGAGTAGCATGTT	
Probe	ACCCCTCGGAAGGTACGCCCTCTTATC	
*FASN*		
Forward	GCTGGCCTACACGCAGAG	NM_001099930
Reverse	GGCCCTGGAGCGGTATCA	
Probe	CGCCTCCAGCACCTTGCCTTGC	
*aP2*		
Forward	CAGGAATTTGATGAAGTCACTGC	NM_001002817
Reverse	GTGGTTGTCTTTCCATCCCAC	
Probe	TGACAGGAAAGTCAAGAGCACCATAACCTT	
*ADIPOQ*		
Forward	CACCACTGGCAAATTCCACTG	NM_214370
Reverse	CCTTCACATCCTTCAAGTAGACC	
Probe	CCTGGGCTGTACTACTTCTCCTTCCACG	
*CD36*		
Forward	CCTACTGGCTGAGTTATTGTGAC	NM_001044622
Reverse	CACAGCATAGATTGACCTGCAA	
Probe	TGGTACAGATGCAGCCTCATTTCCACCT	
*SCD1*		
Forward	ACGGATATCGCCCTTATGACAAG	NM_213781
Reverse	CGCTGGCAGAATAGTCATAGGG	
Probe	TGGAAGCCCTCACCCACAGCTCCC	

### Western blot analysis

Nuclear and cytoplasmic proteins were prepared by using the CelLytic NuCLEAR Extraction Kit (Sigma-Aldrich), according to the manufacturer’s instructions. The protein content was determined by using a bicinchoninic acid protein assay (Pierce, Rockford, IL, USA). Fifteen micrograms of nuclear or cytoplasmic protein were separated by electrophoresis through 12.5 % SDS-polyacrylamide gels (ATTO, Tokyo, Japan). After they were electro-transferred onto nitrocellulose membranes by using the iBlot gel transfer system (Invitrogen), the proteins underwent Ponceau S staining (Sigma-Aldrich) to verify equal loading of the lanes. The membranes were then blocked overnight at 4 °C in phosphate buffered saline (PBS) containing 5 % skim milk and 0.1 % Tween 20, followed by a 1-h incubation at room temperature with primary polyclonal antibodies specific to EBF1 (Abcam, Cambridge, UK), the transcriptionally active form of SREBF1 in the nucleus (Abnova, Aachen, Germany), and β-actin (ACTB) (AnaSpec, San Jose, CA, USA). Horseradish peroxidase-conjugated anti-rabbit antibodies (GE Healthcare, Buckinghamshire, UK) served as secondary antibodies. Antigen–antibody complexes were visualized by use of the ECL detection system (GE Healthcare), and the chemiluminescence signal was scanned with a LAS-3000 imaging system and quantified with Multi Gauge ver2.0, with which the imaging system was equipped (Fujifilm, Tokyo, Japan).

### Lipid accumulation

At each time point of the culture period, PSPA were washed with PBS, fixed with 10 % formaldehyde, and stained with filtered oil-red O solution (0.3 % oil-red O in 60 % isopropanol). The triglycerides in the PSPA and blood from the crossbred gilts were extracted with chloroform–methanol (2:1, v/v) and quantified enzymatically by using the Triglyceride E Test (Wako).

### Data analysis

Measurements of gene expression, protein expression and triglyceride (TG) content were repeated in three independent experiments. Differences in gene expression, protein expression, and TG content among the cell culture treatments were analyzed with Tukey’s multiple comparison tests (*P* < 0.05). Body weight, gene expression levels, BF thickness, and blood triglyceride concentration were compared between crossbred gilts by using the Student *t* test (*P* < 0.05).

## Results and discussion

### Characterization of the full-length sequence of the porcine *SREBF1* gene

First, we determined the 20,099-nt sequence of the full-length porcine SREBF1 gene including the 5′-untranslated region (UTR), the 19 exons that encode the 3,456-nt open reading frame, the 18 introns, and the 3′-UTR (GenBank ID: AB686492). Unlike the human SREBF1 gene (GenBank ID: NG_029029.1), the porcine SREBF1 gene sequence that we determined, together with NCBI reference sequence (RefSeq) information, revealed that there is only one SREBF1 isoform, and no transcriptional variants have been identified in the pig genome (GenBank ID: NM_214157.1) or in cattle [20]. Porcine SREBF1 shares 86 and 83 % homology to the human SREBF1c mRNA and amino acid sequences, respectively. In the full-length porcine SREBF1 gene sequence which we determined in this study, intron 16 included a sequence that is highly conserved among mammals and contains a pre-miRNA sequence (GenBank ID: AB686493) (Supplementary Fig. 1), while the corresponding region in recently published porcine genome sequence (NW_003540979) is not identified. The pre-miRNA sequence expressed mature porcine miR-33b (ssc-miR-33b), which shared high homology with several other mammalian species except for mouse (Supplementary Fig. 1).

### Effect of miR-33b on lipid accumulation in PSPA

**Fig. 1:**
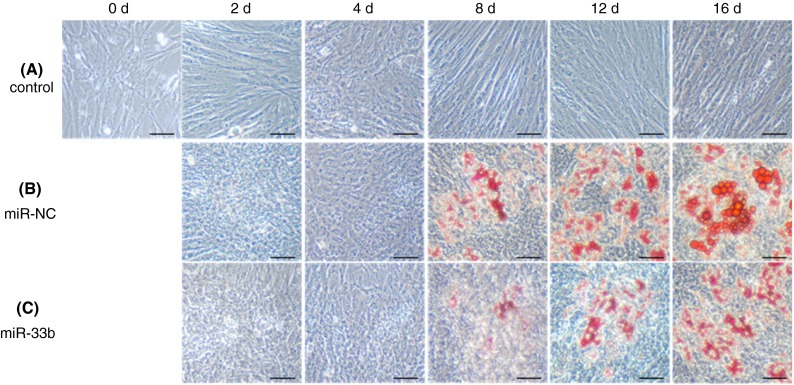
Morphological characterization of PSPA. PSPA were treated with: **a** growth medium; **b** differentiation medium with negative-control miRNA (miR-NC) transfection; **c** Differentiation medium with miR-33b transfection. Lipid accumulation was visualized with oil-red O staining. *Scale bar* 50 μm

**Fig. 2:**
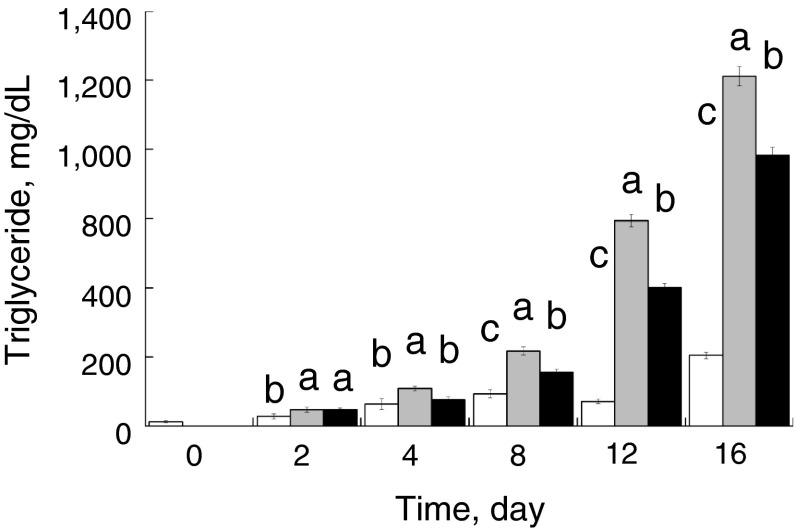
Differences in triglyceride content in PSPA. PSPA were treated as follows: growth medium (*white*), differentiation medium with negative-control miRNA transfection (*shaded*), and differentiation medium with miR-33b transfection (*black*). Triglyceride content was measured at least three times. Comparisons of relative gene expression were performed by using Tukey’s multiple comparison tests. *Different letters* show a significant difference (*P* < 0.05)

To investigate the effect of miR-33b on lipid homeostasis, we transfected miR-33b into PSPA, which represent an ideal experimental model to study porcine adipocyte physiology [21]. Non-transfection/non-differentiation-induced control cells continued to proliferate and showed no signs of morphological change or lipid accumulation throughout the time course, whereas differentiation induction clearly induced lipogenesis after 4 days (Fig. 1). Transfection of miR-33b at differentiation induction decreased lipid accumulation in PSPA after 8 days compared with transfection of miR-NC (negative control microRNA), suggesting that miR-33b affected the differentiation and development of the PSPA (Fig. 1). Consistent with the observation of lipid accumulation by oil-red O staining, the TG content of the miR-33b-transfected cells was significantly (*P* < 0.05) decreased by 70.3 % (4 days), 71.4 % (8 days), 67.6 % (12 days) and 77.4 % (16 days) compared with that of the miR-NC-transfected cells (Fig. 2). These results clearly demonstrate that miR-33b attenuates the differentiation and development of PSPA.

### MiR-33b target gene prediction

To investigate the biological function of miR-33b in PSPA, we explored putative miR-33b target genes by using algorithms including PITA [22], TargetScan v6.0 [23], and MicroRNA.org [24]. These computational predictions suggested that miR-33b may target porcine early B-cell factor 1 (*EBF1*) mRNA, because multiple possible miR-33b recognition sites were found in the 3′-UTR of porcine *EBF1* mRNA, one of which was 100 % identical to that of numerous other mammalian species (Supplementary Fig. 2a, b). In contrast, none of the master regulators of adipocyte differentiation and development were predicted to be miR-33b targets with these methods. These findings suggest that ssc-miR-33b has a conserved effect on the post-transcriptional regulation of EBF1 expression in porcine adipocytes. The BioSystems Database describing “Transcriptional Regulation of White Adipocyte Differentiation” in the NCBI [24] suggested that EBF1 binds and activates the *PPARγ* promoter (http://www.ncbi.nlm.nih.gov/biosystems?term=205243). In addition, Jimenez et al. [26] reported that EBF1 promotes adipogenesis by inducing the expression of the *C/EBPα* and *PPARγ1* promoters, and repressing GATA-2, which is considered to negatively affect adipogenesis in mice [4]. Like SREBF1, C/EBPα and PPARγ are master regulators that are recognized as molecular markers of adipocyte differentiation and development [27–29].

Therefore, we assumed that the observed decrease in lipogenesis in PSPA was caused by ssc-miR33b via the attenuation of adipogenic and lipogenic pathways that are regulated by C/EBPα and PPARγ, which are ordinarily activated by EBF1. We examined the promoter regions of the porcine *PPARγ1*, *γ2*, and *C/EBPα* genes by using TFSERACH ver1.3 based on TRANSFAC [28] and found that the promoters of *C/EBPα* and *PPARγ* genes contained EBF1 and C/EBPα binding sites, respectively (Supplementary Figs. 3, 4, 5). These results suggest that porcine EBF1 may be associated with the transcriptional regulation of C/EBPα and, indirectly, of *PPARγ* genes.

### Effect of miR-33b on SREBF1 and EBF1 expression

**Fig. 3:**
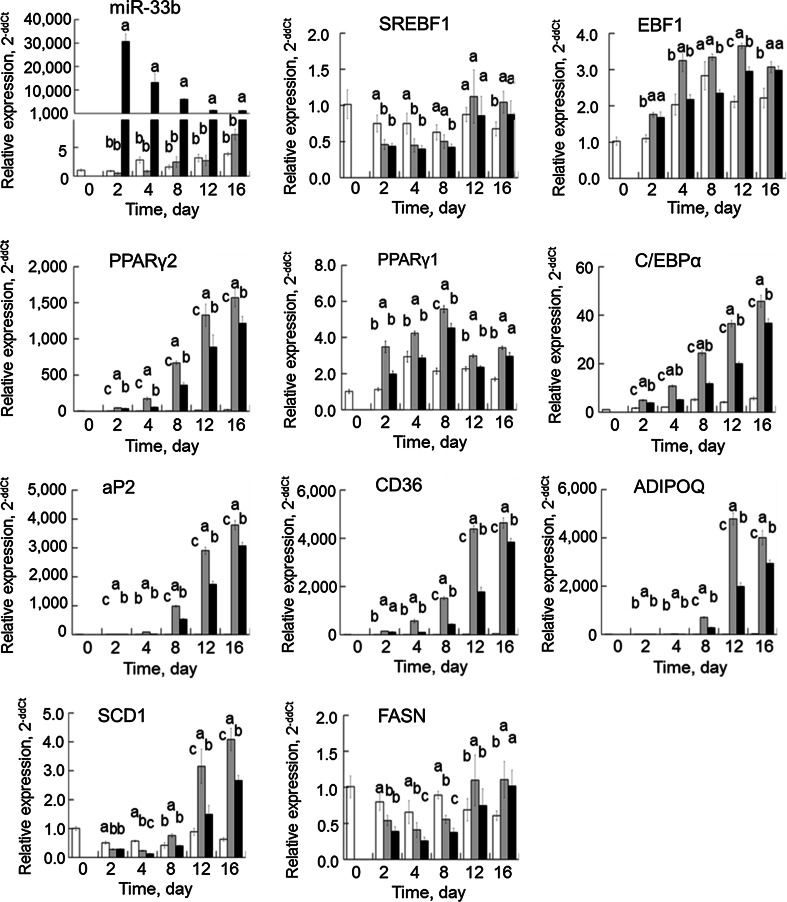
Changes in adipogenic and lipogenic genes in PSPA. PSPA were treated as follows: growth medium (*white*), differentiation medium with negative-control miRNA transfection (*shaded*), and differentiation medium with miR-33b transfection (*black*). Relative gene expression was measured by using the 2^−ΔΔCt^ method at least three times. Comparisons of relative gene expression were performed by using Tukey’s multiple comparison tests. *Different letters* show a significant difference (*P* < 0.05)

Transfection of precursor miRNAs into PSPA successfully induced the transitional expression of miR-33b. The expression level of miR-33b in miRNA precursor-transfected PSPA was significantly higher (*P* < 0.05) than those in the control and in miR-NC-transfected PSPA, although the endogenous miR-33b level was relatively low and did not fluctuate over time (Fig. 3).

**Fig. 4:**
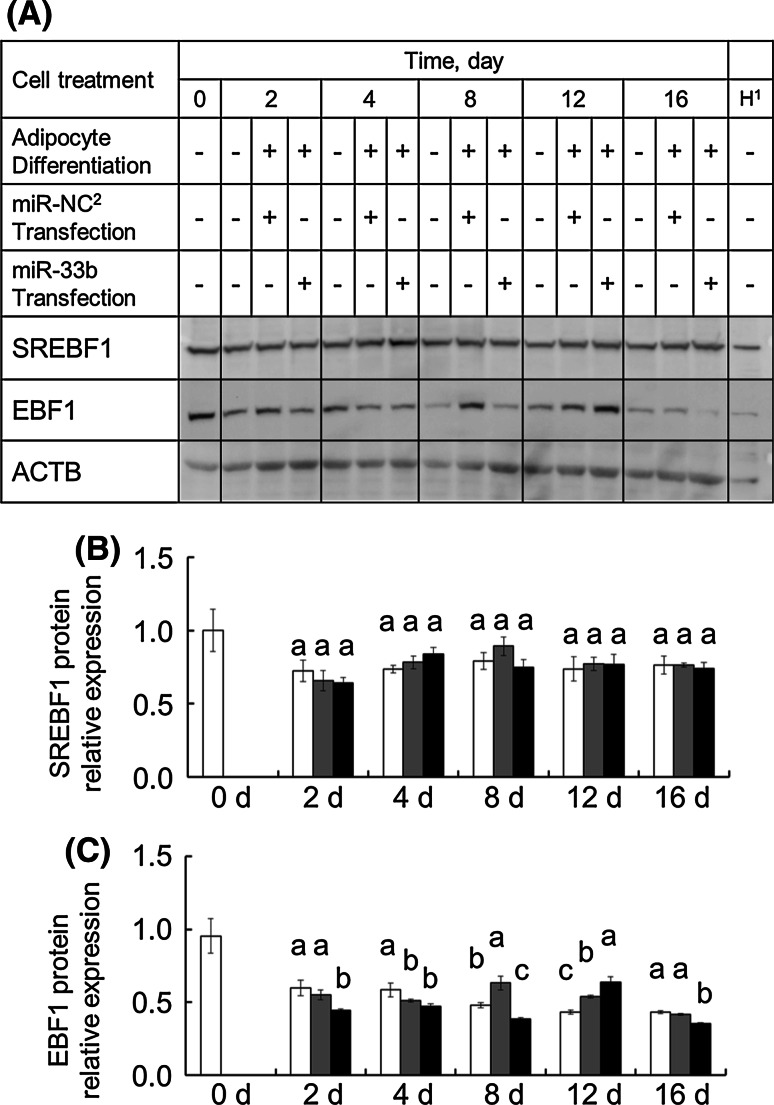
Changes in SREBF1 and EBF1 protein expression levels in PSPA. PSPA were treated as described above. Relative gene expression was determined by Western blotting at least three times. The image shown is a representative result. Comparisons of relative protein expression were performed by using Tukey’s multiple comparison tests. ^1^H: Positive control (nucleoprotein extracted from HeLa cells). ^2^miR-NC: negative control miRNA. *Different alphabets* show a significant difference (*P* < 0.05)

Because of the genomic localization of ssc-miR-33b, we examined whether miR-33b influences its host *SREBF1* gene. *SREBF1* gene expression did not appear to be elevated during the PSPA differentiation induction time course. The mRNA level of *SREBF1* upon miR-33b transfection tended to be lower throughout the time course, but no significant differences were detected (Fig. 3). In addition, the relative protein expression level of SREBF1 after miR-33b transfection was not different from that after transfection of miR-NC (Fig. 4). These results suggest miR-33b had relatively little effect on SREBF1 expression.

In contrast, the mRNA levels of *EBF1* in cells with miR-33b transfection were significantly (*P* < 0.05) decreased by 66.9 % (4 days), 70.2 % (8 days), and 80.8 % (12 days) compared with those after miR-NC transfection (Fig. 3). In addition to this mRNA abundance, the protein expression level of EBF1 in miR-33b-transfected PSPA was significantly (*P* < 0.05) decreased by 80.8 % (2 days), 61.2 % (8 days), and 84.8 % (16 days) compared with that after miR-NC transfection (Fig. 4). These results suggest that miR-33b affects EBF1 expression at both the transcriptional and post-transcriptional levels.

### Effect of miR-33b on *C/EBPα*, *PPARγ* and their downstream adipo/lipogenic genes

Given our finding of decreased EBF1 expression upon miR-33b transfection, we investigated the gene expression of the adipogenic and lipogenic master regulators *C/EBPα* and *PPARγ* and their downstream genes, including *aP2*, *CD36*, *ADIPOQ*, *SCD1*, and *FASN* (Fig. 3).

The relative mRNA level of *PPARγ2* gradually increased in the differentiation-induced PSPA. However, the *PPARγ2* mRNA level after miR-33b transfection significantly decreased compared with that after miR-NC transfection from the 2-day time point onward (*P* < 0.05). In agreement with the *PPARγ2* mRNA expression, the relative gene expression of *PPARγ1*, another isoform of *PPARγ* broadly expressed in various tissue types, was also decreased in miR-33b-transfected PSPA compared with that in miR-NC-transfected PSPA (*P* < 0.05). Similarly, the relative gene expression of *C/EBPα* in miR-33b-transfected PSPA was significantly decreased compared with that in miR-NC-transfected PSPA from the 2-day time point onward (*P* < 0.05). These results demonstrate that the changes in the expression patterns of *PPARγ* and C/*EBPα* were similar, although the levels of gene expression were different. The results of the gene expression assays for *PPARγ2* and *C/EBPα* suggest that the decrease in expression of these genes was affected by EBF1 degradation, as indicated in the BioSystems Database of the NCBI and by Jimenez et al. [26].

Genes known to be regulated by PPARγ, such as *aP2*, *CD36*, and *ADIPOQ*, were highly expressed in adipose differentiation-induced PSPA, whereas their expression was significantly decreased in miR-33b-transfected PSPA (Fig. 3). In addition, the same fluctuating expression pattern was observed for the *PPARγ2* gene, suggesting that the decreased expression of the *aP2*, *CD36*, and *ADIPOQ* genes was the result of decreased PPARγ2 in miR-33b-transfected PSPA. In human adipocytes, transcription of *aP2*, *CD36*, and *ADIPOQ* is reported to be regulated by PPARγ2, and these genes are associated with fat formation and metabolism [31–33].

The mRNA level of *SCD1* was generally increased in line with the degree of lipogenesis in adipose differentiation-induced PSPA, but the mRNA level after miR-33b transfection was at least 30 % less than that after miR-NC transfection from the 4-day time point onward (*P* < 0.05) (Fig. 3). The observed decrease in *SCD1* mRNA expression levels was similar to that of the *PPARγ2* mRNA level, because *SCD1* gene transcription can be regulated by SREBF1 or PPARγ2 [5, 34]. The mRNA level of *FASN* after miR-33b transfection was 41 % less than that after miR-NC transfection at 4 days (*P* < 0.05). In contrast to the *SCD1* mRNA level, changes in the *FASN* mRNA level were small, suggesting that *FASN* transcription may be modestly regulated by PPARγ2, as demonstrated in previous studies [35, 36].

Taken together, these results suggest that expression of adipogenic and lipogenic genes is well regulated by the master regulators PPARγ2 and C/EBPα in PSPA and that the observed decrease of lipid accumulation in PSPA can be explained by the decrease in expression of these key genes. Furthermore, the delay in adipogenesis and decrease in lipogenesis observed in PSPA after miR-33b transfection was characterized by the degradation of PPARγ2 and C/EBPα but not that of SREBF1, possibly due to decreased EBF1 transcriptional regulation.

### Possible effect of miR-33b on fat formation differences between pig breeds

To investigate the effect of miR-33b on fat formation in fattening pigs and on the generation of different fat traits in pigs that were crossbred with typical lean (Landrace)- and fatty (Meishan)-type breeds, we analyzed differences in BF thickness and blood TG levels and also in miR-33b, *SREBF1*, *EBF1*, *PPARγ*, and *C/EBPα* gene expression in the subcutaneous tissues of EL and EM crossbred gilts.

**Fig. 5:**
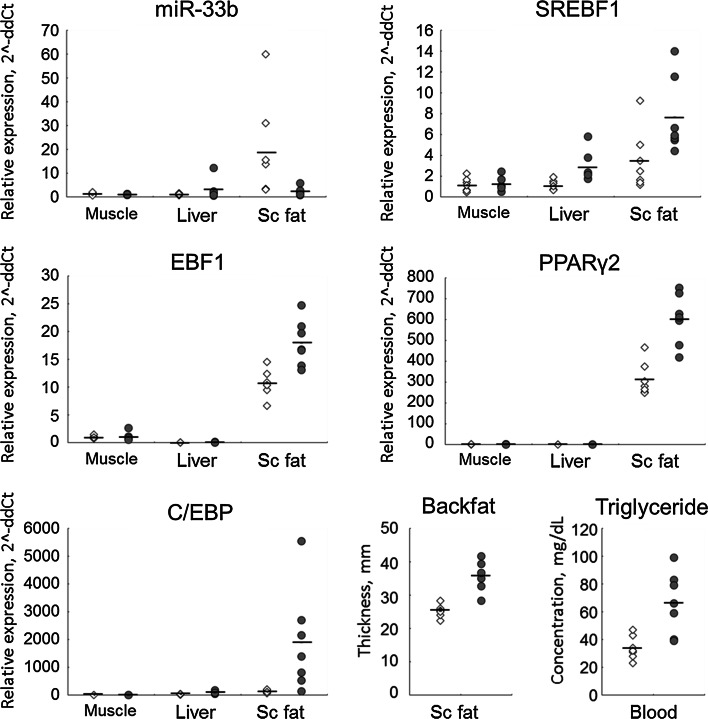
Comparison of miR-33b, lipogenic genes, and fat formation indices between Meishan- and Landrace-derived crossbred gilts. Expression of miR-33b and lipogenic genes in muscle (LD muscle), liver and Sc fat (subcutaneous fat) tissues. *Open squares* and *shaded circles* indicate individual crossbred gilts derived from Landrace (EL) and Meishan (EM), respectively. Mean values are denoted with *horizontal bars*

We found that EM gilts showed significantly higher blood TG levels (*P* < 0.01) and BF thickness (*P* < 0.001) than did EL gilts, in agreement with a previous finding [37]. In addition to this difference in physiological characteristics between these pig breeds, gene expression assays indicated that miR-33b expression in EL tended to be higher than that in EM (*P* = 0.08) (Fig. 5). In contrast, EL gilts showed significantly lower expression of lipogenic genes, including *SREBF1* (*P* < 0.01), *EBF1* (*P* < 0.01), *PPARγ2* (*P* < 0.01), and *C/EBPα* (*P* < 0.05) than did EM gilts. These results suggest that the difference in miR-33b expression between these pig breeds is associated with differences in the transcriptional regulation of lipogenic genes, leading to observed differences in fat-related traits such as BF thickness and blood TG levels. It is important to note that only seven gilts were used to obtain these data; therefore, given the small sample size, it is difficult to draw conclusions regarding correlations between miR-33b and lipogenic gene regulation and fat-related traits. In addition, genetic polymorphisms in *SREBF1* and miR-33b may have certain effect on their expressions, although so far there is no evidence for a difference in the *SREBF1* gene sequences between Landrace and Meishan pigs. As described in a review by Dodson et al. [2], meat animals have recently begun to be utilized as good experimental models for lipid metabolic research. In addition, the lack of miR-33b in *Srebf1* gene in traditional model animals such as mouse and rat (Supplementary Fig. 1) [11, 12] may mean that the pig is a better model for biomedical research on lipid metabolism.

Future studies of miR-33b will include evaluating correlations between miR-33b, lipogenic genes, and pork carcass characteristics related to fat tissues by using a larger number of pigs.

## Electronic supplementary material

Below is the link to the electronic supplementary material.

Supplementary material 1 (DOCX 40 kb)

Supplementary material 2 (DOCX 26 kb)

Supplementary material 3 (DOCX 25 kb)

Supplementary material 4 (DOCX 18 kb)

Supplementary material 5 (DOCX 25 kb)
