# Supplementary material for: MicroRNA-33b downregulates the differentiation and development of porcine preadipocytes
Source: Mol Biol Rep. 2014 Jan 8;41(2):1081–90. doi: 10.1007/s11033-013-2954-z (PMC3929038; doi:10.1007/s11033-013-2954-z)
Supplement: Supplementary file 1 — Supplementary material 1 (DOCX 40 kb) [file 11033_2013_2954_MOESM1_ESM.docx]

10 20 30 40 50 60

....|....|....|....|....|....|....|....|....|....|....|....|

**Human GCAGCTCCATTGACAAGGTGAGGGGTGGGGTCAGGGGCCTGGCAGGGCT----GGGG---**

**Chimpanzee GCAGCTCCATTGACAAGGTGAGGGGTGGGGTCAGGGGCCTGGCAGGGCT----AGGG---**

**Rhesus monkey GCAGCTCCATTGACAAGGTGAGGGGTGGGGCCAGGGGCCTGGCAGGGCT----GGGG---**

**Pig GCAGCTCCATTGACAAGGTGAGGGGTGGGGCTGGGGGCCCGGCAGGTCTG---GGGGGGG**

**Cow ACAGCTCCATTGACAAGGTAAGGGCTGGGGCCAGGGGCCTGGCCTGTCTC---AGGG---**

**Cat GCGGCTCCATTGACAAGGTGAGGGTTGGTGCCGGGGGCCTCGCAGGTCT----GGGG-GG**

**Dog GGAGCTCCATTGACAAGGTGAGGGGTGGTGCCGGGGGCCTGGCAGGTCT----GGGGTGG**

**Horse GCAACTCCATTGACAAGGTGAGGGGTGGGGCCAGGGGCCTGGCAGGTCTGTGGGGGGGGG**

**Mouse GCAGTTCCATTGACAAGGTGAGGGGTAGGGT-GGGTTCCTGTCTAGGCC----AGGG---**

**Rat CCAGTTCCATTGACAAGGTGAGGGGTAGGGT-GGGTTCCTGTCTAGGCC----AGGA---**

70 80 90 100 110 120

....|....|....|....|....|....|....|....|....|....|....|....|

**Human --GATTCAGCTTT-CCATTCCCTGGTTCCTCTCCCCAGCCCCCAGGGGCTGCAGAAGACC**

**Chimpanzee --GATTCAGCTTT-CCATTCCCTGGTTCCTCTCCCCAGCCCCCAGGGGCTGCAGAAGACC**

**Rhesus monkey --GACTCAGCCTT-CCATTCCCTGGTCCTTCTCCCCAGGCCCCAGGGACTGCAGAAGACC**

**Pig GGGTCGAGGCCTTTCCATTCCCTGGATGGTCCCT-T--------GGGGCTGCAGAAGACT**

**Cow --GCCTTGGCCTTTCCACTCCCTGGATGGTCCCCCT--------TGGGCTGCAGAAGACT**

**Cat CGGTCGCCGCCTTTCCACTCCCCGGACGTTCTTCCT--------TGGGTTGCCGAAGACT**

**Dog GGGTCCCCACCTTGCCATCCCCTGGATGTTCTCCCT--------TGGGCTGCAGAAGATG**

**Horse CGGTCACAGCCTTTCCACTCTCTGGATTTTCCCCCT--------TGGGCTGCAGAAGACT**

**Mouse ---TCACAGCTGT-CCATTTCCAGG---TTCT----------------------------**

**Rat ---TCACAGCTAC-CCATTCCCAGT---TTCT----------------------------**

130 140 150 160 170 180

....|....|....|....|....|....|....|....|....|....|....|....|

**Human ATGGGG-TTAGCCCAAG-CAGCACAGGATAGGGGGTCCAGCAGA----------------**

**Chimpanzee ATGGGG-TTAGCCCAAG-CAGCACAGGATAGGGGGTCCAGCAGA----------------**

**Rhesus monkey ATGGGG-TTAGCCCAAG-CAGCACAGGATGAGGGGTCCAGCAGATAGGAGATCCAGCAGT**

**Pig GCAGGGGTCGGCCCAG--CAGCACAGAGCTGGGGGGCTAGTGGG----------------**

**Cow GCAGGG-TCAGCCCAA--CAGCACAGGACGGGGGACCCAGCCGG----------------**

**Cat GTGGGG-TTAGCCTGA--CAGTACAGGACTTGAGGCCCGGCTCG----------------**

**Dog CTGGGG-TCAGCCTGA--CAGTAGGGG--TTGGGGCCCGGCCAG----------------**

**Horse GTGGAG-TTAGCCCAA--CAGCACAGGCCTGGGGGTCTAGCTGG----------------**

**Mouse -TGGTG-TTAGTACAGAATAATGCTAAAT---GGATTTA---------------------**

**Rat -TAGAG-TTAGTACAAAATAATGCTGAGT---GGACTTA---------------------**

190 200 210 220 230 240

....|....|....|....|....|....|....|....|....|....|....|....|

**Human CCCTGCTT-TTTGGCTAAGGCTTCTGTCCAGA--GGAGAGGGGTTGCCCCTATCTGGCCT**

**Chimpanzee CCCTGCTT-TTTGGCTAAGGCTTCTGTCCAGA--GGAGAGGGGTTGCCCCTATCTGGCCT**

**Rhesus monkey CCTTGCTT-TTTGGCTAAGGCTTCTGTCTAGA--GGAGAGGGGTTGCCCTTATCTGGCCT**

**Pig CCTTGCTT-TCTGGCTGAGGCTTGGGTCCAGG--GGAGAGAGGCTGCCCCC-TCTGGTCT**

**Cow CCTTGCCT-TCTGGCTAAGGCTTGGGTCCAGG--GGAGAATGGCTGCTCCC-TCTGGCCT**

**Cat CCTTGCTTTTCTGGCTGAGGCTTGGGTCCAGG--GGAAAGTGGCCACCCCT-CC------**

**Dog CCTTGTCTCTCTGGCTAAGGCTTGGGTCCCGGTGGAAGAGCGGCTGCCCCC-CTCGGGTC**

**Horse CCA---------GGCTGAGGCTTGGGTCCAGA--AGAGAACAGCTGACCTT-TCTGGCCT**

**Mouse CCTCAGTT--TTAACGACGGCTTAAGTCTTGG--GGAGAGGGGGCTTTACTGTT------**

**Rat CCTCAGTT-CTTGACGAAGGCGTGAGTCTTGG--GGACAGTGGTCTC-ACTCTT------**

250 260 270 280 290 300

....|....|....|....|....|....|....|....|....|....|....|....|

**Human CAGTTTCCCCATCCCTGGGAGGAGGG---GGGTGGATGGTGTGGTAGGATCCCTTTGGAG**

**Chimpanzee CAGTTTCCCCATCCCTGGGAGGAGGG---GGGTGGATGGTGTGGTAGGATCCCTTTGGAG**

**Rhesus monkey CAGTTTCCCCATTC-TGGGAGGATGG---GGGTGGATGGTGTGGTAGGATCCCTTTGGAG**

**Pig CAGTGTCCCCACCC-CAGGAGCAGGGGCGGGGCAAGCAGTGTGGCAGGGCCCTCTTGGAA**

**Cow CAATGTTGCCCTCC-TAGGAGGAGGG-TGGGGTGAACA-TGTGGCAGGGCCCTCCTTGGA**

**Cat ----GCCCCCGCCCTCAGGAGGAGGGGTGGGGTGAGTGGTCTGGCAGAGCCCTCTTTGGA**

**Dog CCA-GTCTCCCCCCACAGGAGGACGGGTGGGGTGAGGGGTCTGGCAGAGGCCTCTTCCGA**

**Horse CAGTTTCCCCACCCCCAGGAGGAGGGGTGG--TGAGTGGTGTGGCAGGGCCCT-------**

**Mouse -----TCGCCACCACCAGGAAGGAGGGTGTGGGGAGCAGACTGGCAGGGTTCCTGTTGGG**

**Rat -----TTGCCACCACCAGGAAGGA---TGTGGAGAGTGGACTGGCAGGGCTCCTGTTGGG**

310 320 330 340 350 360

....|....|....|....|....|....|....|....|....|....|....|....|

**Human GCCCTGCATCAGGAGGGCTGGACAGCTGCTCCCG-GGCCGGTGGCGGGTGTGGGG-----**

**Chimpanzee GCCCTGCATCAGGAGGGCTGGACAGCTGCTCCCG-GGCCGGTGGCGGGTGTGGGG-----**

**Rhesus monkey GCCCTGCATCAGGAGGGCTGGACAGCTGCTCCTG-GGCCGGTGGCGGGCGTGGGG-----**

**Pig G-----------------------GCTGCTCCAGAGGCAGGTGG-TGGCATGGTG-----**

**Cow G-----------------------GCTGCCGCGCAGGCCGGTGG-GGGCATGGGG-----**

**Cat G-----------------------GCTGCCCCTGTGGCAGGCGGCAGGTGTGGGG-----**

**Dog G-----------------------GCCACTTCTG-AGCGGGCG-CAGGCGTGGGG-----**

**Horse G-----------------------GCTGCTCCTGTGGCAG-TGGCAGGTGCGGGG-----**

**Mouse A-----------------------GCCATTGTTG-GGAAGACTGCGGGCTTGTAGCA---**

**Rat G-----------------------GCCATCGTCA-GGAAGAGGGAGGGCTTGTAGCATGT**

370 380 390 400 410 420

....|....|....|....|....|....|....|....|....|....|....|....|

**Human -GCCGAGAGAGGCGGGCGGCCCCGCGGTGCATTGCTGTTGCATTGCACGTGTGTGAGGCG**

**Chimpanzee -GCCGAGAGAGGCGGGCGGCCCCGCGGTGCATTGCTGTTGCATTGCACGTGTGTGAGGCG**

**Rhesus monkey -GCCAAGAGAGGCGGGCGGCCCCGCGGTGCATTGCTGTTGCATTGCACGTGTGTGAGGCG**

**Pig -GCCTGGAGAG-CGGGCGGCCCCGCGGTGCATTGCTGTTGCATTGCACGTG--TGAGGCG**

**Cow -GCCTGGAGAG-CGGGCGGCCCCGCGGTGCATTGCTGTTGCATTGCATGTG--TGAGGCA**

**Cat -GCCTGGAGAGGCGGGCGGCCCCGCGGTGCATTGCTGTTGCATTGCACATGAGTGAGGCT**

**Dog -GCCTGAAGAGGCGGGCGGCCCCGCGGTGCATTGCTGTTGCATTGCACGTGAGTGACGCG**

**Horse -GCCTGGAGAGGCGGGCGGCCCCGCGGTGCATTGCTGTTGCATTGCACGTGTGTGAGGCG**

**Mouse --------GGA---GGCGG-------------TGCA--TGTGTTG-AGGC-----A----**

**Rat AGCCTAGAGAA---GGTGGGCCTGTGACACACTGCACTTGCACTGCACGTC--TGA----**

430 440 450 460 470 480

....|....|....|....|....|....|....|....|....|....|....|....|

**Human GGTGCAGTGCCTCGGCAGTGCAGCCCGGAGCCGGCCCCTGGCACCACGGGCCCCCATCCT**

**Chimpanzee GGTGCAGTGCCTCGGCAGTGCAGCCCGGAGCCGGCCCCTGGCACCACGGGCCCCCATCCT**

**Rhesus monkey GGTGCAGTGCCTCGGCAGTGCAGCCCGGAGCCGGCCCCTGGCACCGCGGGCCCCCATCCT**

**Pig GGTGCAGTGCCTCGGCAGTGCAGCCCGGAGCCGGCCCCTGGCACCGAGGGCCCCC-ACTC**

**Cow GGTGCAGTGCCTCGGCAGTGCAGCCCGGAGCCGGCCCCTGGCACCGCGGGCCCCCTAGCC**

**Cat GGTG--------------------------------------------------------**

**Dog GGTGCAGTGCCTCGGCAGTGCAGCCCGGAGCCGGCCCCTGGCACCGCGGGCCCCCACTCG**

**Horse GGTGCAGTGCCTCGGCAGTGCAGCCCGGAGCCGGCCCCTGGCACCGCGGGCCCCCACCCC**

**Mouse GATGCAGTGCTTCAGTGATGCAACCTGGAGTCAGGCCCCAGTGCTGTGGACTTCCTGCCC**

**Rat GGTAGAGTGCTTCAGTGACGCAGCCTGGAGTCAGGCCCTGGCGCTGTGGACTTTCTGACT**

490 500 510 520 530 540

....|....|....|....|....|....|....|....|....|....|....|....|

**Human GCCC-----CT-------------------------------------CCCAGAGCTGG-**

**Chimpanzee GCCC-----CT-------------------------------------CCCAGAGCTGG-**

**Rhesus monkey GCCC-----CT-------------------------------------CCCAGAGTTGG-**

**Pig CTCC-----CT-------------------------------------CCCCCGAGCCG-**

**Cow TTCT-----CT-------------------------------------CCCACAGCCAG-**

**Cat ------------------------------------------------------------**

**Dog TCCC-----CT-------------------------------------CCTACAGCCGG-**

**Horse TACC-----CT-------------------------------------CCCAGAGCTGG-**

**Mouse TTACTGACGCT--------------------GCCTTGAGACAGGGTGGCCCTCAGCTAGG**

**Rat TTTCCAATGCTAGAGCCTCCCAGGGGTGCAGGCCTTGAGATAGCTTGGCCCTCAGCTAGG**

550 560 570 580 590 600

....|....|....|....|....|....|....|....|....|....|....|....|....|

**Human ---AGCCC-----TGGTG-----ACCCCTGCC-CTGCCT-----GCCACCCCCAGGCCGTGCAG**

**Chimpanzee ---AGCCC-----TGGTG-----ACCCCTGCC-CTGCCT-----GCCACCCCCAGGCCGTGCAG**

**Rhesus monkey ---AGCCC-----TGGTG-----ACCC-TGCC-CTGCCC-----GCCACCCCCAGGCCGTGCAG**

**Pig ---ACGCC-----CCCTG-----ACCCCTGCC-CTGCCCTGCCCACCACCCCCAGGCCATGCAG**

**Cow ---AGGCC-----C--TG-----ACCCCTGCC-CTGCCCTACCCACCACCCCCAGGCCTTGCAG**

**Cat ----------------------------------------------------------------**

**Dog ---AGCCCA-CCCCCGTG-----ACCCCTTCC-CTGCCC------CCCACCCCAGGCCATGCAG**

**Horse ---AGCCC----CCCATG-----ACCCCTGCC-CTGCCC-----ACCACCCCCAGGCCATGCAG**

**Mouse GAAAGCTTGATGTCCATAGGGAAACTCCTCCCTCTGCCCTGCCTGCTA-TCCCAGGCCATGCAG**

**Rat GAAAGGCTGATTTCCATAGAGAAATTCCTCCCTCTGCCCTGCCTGCTA-TCCCAGGCCATGCAG**

Supplementary Figure 1

Alignment of the nucleotide sequence of intron 16 in *SREBF1* among mammals

Mature miR-33b sequences are highlighted in yellow.
