# Supplementary material for: MicroRNA-33b downregulates the differentiation and development of porcine preadipocytes
Source: Mol Biol Rep. 2014 Jan 8;41(2):1081–90. doi: 10.1007/s11033-013-2954-z (PMC3929038; doi:10.1007/s11033-013-2954-z)
Supplement: Supplementary file 2 — Supplementary material 2 (DOCX 26 kb) [file 11033_2013_2954_MOESM2_ESM.docx]

(A)

1 AAGAAUUGCCUUGAAGAAUUUUAUUAAUGAAAAGGUUGGAUUCUGCUACAGAGAGUAAUC

61 UGAUACAAGUCCCAGAGUGGAACUUUUAACUCAGGCCUUUUUAAGAGGAAUCACAAUAAC

121 UGCAGAUUUUUAAACAAACAUUAUCACCGACCUUGCAAAUACUGAAAUUGGAAGGGAUCU

181 GCGAAUGCAGGGUGUUGGUUACAGUUGUACCUCCCGAGUCCUUGGGGGGAUAUAUUUAUU

241 CCGUGUUGAUAAAAGCAAAUCCACUUUUCCUUUUCUUUUUUUUUUUUUUUUUUUUUUAAG

301 CUUAACUGCAAUCAUUUGUCUUUUAUAAACCGUAAAGCUGUAUACAAGGGACACUAUAAA

361 UAAGACUCCAUGUUUUAAUUUAUGAUGUUUUUAAAGCUGUGUAAAAGGAGAAUGAAGUGG

421 UGAUAUUUACAAAAAAGUUAAAAAAAAGAAAAAAAAAGAAAAAAGAAAAAAAAAGCUUGU

481 AUGGGACAGAAUAGGAAUGCCAGUUAGAUUUUUUAGAAAACUAAGGGUCGGCUUUUGCGC

541 CUUAAAGCAUAUCAAGUGGUAGUUACUCGGACAGUGCAUUUCCAGUAUCUAACUUAACAC

601 GCCAUCCCUUAGCAGUGCAAGCUUAUUUAUCUCUUUUGUAUUGUUGUCUUAAGUAACUGU

661 GUAAAUAAAUGCAGCCUGGAAAGUUAAAAAGUGAUGUAAGUGAUCACAGUUCCCCUUCUG

721 CUCAAAAAUCCAGUGCCUCUAGACAGAUGUUACAACUGCAUAUUUAAACCUGAUAUCACU

781 CUCUCUCUUGCUUAUGUCAACUUCUUCUGAAGCCGGUGGCCUCUUGAGAGCUUGGUGGCA

841 CACACAUUUGUGAGGAUCUCCUUUGAGACUUCAUGGAACAGGGUCAGGCACAGAAUUCCA

901 CAUAUGCCCUCCAGUUAUCAAGCCAAAAUCCCACUUACACGUCGCCAUUGACAAGUUUAA

961 AGUUUACUCAUUCUAACUGCGGACUUCAUACCAAUCCUCAAAAGCUGACAAAAGGAACGU

1021 GACAGGUAUUUCAGAUGACCCAUUCGAGACAGCGCCUCUAUCAGUCGACCCUUAACCAUU

1081 UGUAACAUAGUAGAUUAAAACGACAGGUUAAGUGCUUUGGAAUUAAGAAUAAAAGGAAAC

1141 UGGGAGAAAGGAGAAGGUUAAGACCUCCGAAACAUAGUUUUCUGUUCGAUGGGAAUUUUU

1201 GCUCUCAUUAUCCGGGAAGUGUUCUUAAAAAUAGGAAUUAAUAGCAGAGAUGCAGCAAAG

1261 CUCUGAGGAUGCAUUUGCCUGGUAUUUUUUUCUUUGCUGUUGUGUUUUUGUAUGUAGUUA

1321 UAAAUACUGUAGAUUUUUUGUGAUUUUUUGCCAAAGUUGUGGUUCUAUUUAUACAUUUUA

1381 AUGUCUUAAGACAGUUUUUCAAUAUCACACACAAAAAAGAUUUUACUGCAUAUUUUGCAA

1441 AGAAAAAAAAGCUCACUACCUUUAGCUUGCACAUACUUGCAAAGUUAAUUAAAAGGCUUU

1501 UUGUUUUAAAGGGGAUUUUGUAAGAUAUCCAUAUAAAUAAUGUAUUUAUCUUUGGAAUUU

1561 GUACAUUGCUUUCCCCUUCCUUCUUUUCCUCCGACUCCCAAUUUAUUUUAUUGUGUAUGU

1621 UUGCUAUGUGAAAAGUGCGUAUUGGUUUGGUCACCUAUAGUUGUAUUAGCUGUUUCAAUG

1681 UGAUUUUUAAACAUUUCAUUUAUAGUUAUUUUUAGUAUUGUUUUAAACCAUGCUUCAUUU

1741 UUUAAUUUUCAUCCAAAAGCCAUUGUCUAUUUUUGUAUUAUUUGUAAGUUAAGAAGUUUU

1801 UUCCAAUAUAUGGCAAAAAAAUAGUAGCAUAUUAUUCUUGUAGCAUUUAGUUCCGUAGAU

1861 UUAAAAAAAAUGUAUCCUUUGCUUUGGAAGCUUACAGAAAAAAAACAAAAAACAAACAAA

1921 AAAAAACAACCCUAAUGCUGUUUUACUCUAUUAAUAUGCAUGGAACCUCUCCCUUUGGAG

1981 UGACGCAUUUUGUGCAUUAAAUUCCGAGGAGAAACUUCAUAGAAAUCAGUGAACAUACUU

2041 UCUUUCCUAAGUCUGCUUGUAUAUUUCCUCUGUCUUUCACAUAAAUAUAAACCAGCAGAU

2101 UGGAUGCCUUAACAAUGCAAAUCAUAUUCAUUUCACUUGUACAUUGUAACUGUGCACCAG

2161 AACUGUCAGUCAUCACUCACAUUCUAAGAAAAAAGAAAAAAAAAAAAAAGAAAAAAAAAG

2221 AAAUCGAAAAGCACAAAAGAACUGUUUUGUUACCUUAAGACAAUGUAACUUUUCUAGUAG

2281 AGCAAGAAAUUUACAACAAUGCUGCAACUGUGCAUGCCCCCCUAUGGAUUUUGCAAUGGU

2341 UUUCACUAGACUGUCAGAGUGGAUUUUUAUGGGUUGGGGCGGGUGGGGGGAGGGGUUUGC

2401 GGGAGGGAAGGGAGGGGAGGAAAGCUGAUUUUUCUUGGUGAGAAAUAAUAAUAAUGAUGA

2461 AUAAUAAUAAAACUGGAAAAUGUAAGCAAGGUGGACGCAUUGCUUCUCGGUACUCAGAAA

2521 GGUUGUCUGAAUUCGUGUGGUAAGCGCUGGCCUGAAGAUGUGUACAAUUUAAAGCCAUAU

2581 UUUGUCUGGUGAGCCCCUUAACUGUUUGUGAAGGACCGGUCAGCCGGUGAGGUAUCCGGC

2641 UCAGACCCUGACAACAGACGCCACCCAUAGUCAGCCAUGCGUAGUGGUUAGAACUUAUCU

2701 UGAAAGUCCAAAGAGCCUUUAAAAUGUGUAUCAUUUGUGUUUUGUUGCCUCUAUUUAUAU

2761 UGAUUAGAUGAAAAGACGUUCUGACCCUCGGACCCUCUUUCUUCUACACAAAACUUUUAC

2821 AAGUAAAUAUGUUCCCCUAAAUACAGAAUAUGGCUUUAAGACGAAAAUAGAGAAUUCAAC

2881 UAAGAUUUUGGUUUUGGGGGAAAAAUACAGCUUCUGGAUGACUGGAUUGCAUAACAUGCC

2941 CUGGCCUCACAUUGUACAAGGAUGCAGCUUAACAAAGUCAUCUCUAAGUCUAACCUUUCA

3001 CCUUCUUAUCAAACUUUUGGAAUAGACACACACUGUACAGUUCAAUUGUUAGAGAACCUA

3061 ACUACUGUAGAGAUUGUUAUAAUUUUUUUUUUUUGCAAAAAUUCAAGCUGUAAAAACUUU

3121 UCAACUUUCACAAUAUUUAAUUAAAG

(B)

**2081 2091 2101 2111 2121 2131 2141**

**|||||||||||||||||||||||||||||||||||||||||||||||||||||||||||||**

**Pig UAUAAACCAGCAGAUUGGAUGCCUUAACAAUGCAAAUCAUAUUCAUUUCACUUGUACAUUG**

**Cattle UAUAAACCAGCAGAUUGGAUGCCUUAACAAUGCAAAUCAUAUUCAUUUCACUUGUACAUUG**

**Cat UAUAAACCAGCAGAUUGGAUGCCUUAACAAUGCAAAUCAUAUUCAUUUCACUUGUACAUUG**

**Dog UAUAAACCAGCAGAUUGGAUGCCUUAACAAUGCAAAUCAUAUUCAUUUCACUUGUACAUUG**

**Horse UAUAAACCAGCAGAUUGGAUGCCUUAACAAUGCAAAUCAUAUUCAUUUCACUUGUACAUUG**

**Human UAUAAACCAGCAGAUUGGAUGCCUUAACAAUGCAAAUCAUAUUCAUUUCACUUGUACAUUG**

**Chimpanzee UAUAAACCAGCAGAUUGGAUGCCUUAACAAUGCAAAUCAUAUUCAUUUCACUUGUACAUUG**

**Rhesus Monkey UAUAAACCAGCAGAUUGGAUGCCUUAACAAUGCAAAUCAUAUUCAUUUCACUUGUACAUUG**

**Mouse UAUAAACCAGCAGAUUGGAUGCCUUAACAAUGCAAAUCAUAUUCAUUUCACUUGUACAUUG**

Supplementary Figure 2

Predicted target sites of ssc-miR-33b on the porcine *EBF1* gene and EBF1 homology among species.

1. Predicted target sites of ssc-miR-33b on the 3'-UTR of the porcine *EBF1* mRNA were detected by using PITA (denoted by 6 boxes: <http://genie.weizmann.ac.il/pubs/mir07/mir07_data.html>), TargetScan v6.0 (denoted by green highlighting: <http://www.targetscan.org/cgi-bin/targetscan/vert_60/view_gene.cgi?taxid=9606&rs=NM_024007&members=miR-33ab/33-5p&showcnc=0&shownc=0>), and MicroRNA.org – Targets and Expression (denoted by the same green highlighting as for TargetScan: <http://www.microrna.org/microrna/getGeneForm.do>).
2. Alignment of the *EBF1* mRNA 3'-UTR proximal region of the ssc-miR-33b site demonstrating that the predicted target site (yellow highlighting) is highly conserved among mammals. Numbers indicate nucleotides from the start of the 3'-UTR of the porcine *EBF1* mRNA.
