# Supplementary material for: MicroRNA-33b downregulates the differentiation and development of porcine preadipocytes
Source: Mol Biol Rep. 2014 Jan 8;41(2):1081–90. doi: 10.1007/s11033-013-2954-z (PMC3929038; doi:10.1007/s11033-013-2954-z)
Supplement: Supplementary file 3 — Supplementary material 3 (DOCX 25 kb) [file 11033_2013_2954_MOESM3_ESM.docx]

aggaattgggacagggtgtcatggagcgagaaggcctatctttgctcccgatgtttggagcctccgctgaaagtgtcaaaagcgtttcactcacatatctggcagtcagtgctggctcttgaccagggggcttcctttcctcgccatgtggacctctctctgtggcctgggcttcctcacaacgtagtggctgcgttccaagggcaagcatcccaacagaaaaagagccaagtggaagctggaagtcacacagcctcactccatcccgtctcttagaggtgaatcactcgggccagctcctcttccaggggaggagaattaggctccaccttttgggagggaagggtcaaagcctttgtggacatgttttaaaactgtcacaataaccaagtggaattctttgtgataaaactaagaatgtgctatataaacaaaccttaaaatgttaattctacagcaaaatatgatgacatgcgaaacaaagatgcacaggcagacttttaaacaacataaggcatggccatttgtcatgaacaaacgctttgttgagttttgcacgttcccttgtttttccatctcttagatcaggtaaatctattagaatcattattaagaaaacccagccgtttcctgttgacaggagataggaacaaaacaaaatcagaaatcgtggagtgcctgtcgtggctcagcagaaacaaatccgactaatatccatgaggatgcgggttcaatccctgaccttgcttagtaagtcgggggtctgatgtcgctgtggctgtggtggaggccggccgctggagctctgattggacccctagcctgggaacttccacatgctgagggtgaggccctaaaaagaaaaaaaacaaaaagtcaaaagttaaacagagaggattttaaaactttaaaaagagatgtaacaaatacaccagatgctctaagaaaaataattcagggtgaaacgtgaggggattccatcatcactaaccttagagataacccgagaaaatcaaagttaaaataacgagacggcaaacaaagattttcaagtattaataatgttcattgctcgcatgggtgtcaggaagggtgtatgtattcacttgttgggtgtgtgacattgtacacccttttttcaaggataatttgcagtatctgttaaattttaaaccccgcacactttgacccaacagtttcaccgctgagtctccttggcccacacacatctttactaaagcaattctgtgtaaatcctatgcagagttcttaaaaggaagatctattcatggacaatgttcaatgaaaagagcaagcagcaaaacgatacacacagcacgaggcctcttgtatattttaaattatagaaccaaatcatatatctgcaacttgtttatgtccatacatatgctaaacacaaagaaaagcacgaagaaagatttgcattaaaatgataacagtggcttcacaggattaaaagtttggaataagggggaaatttaaaattttttcctctataccctgctaatattttggatttctgaaacataaagtatacatgtagtagctctaccaatttaaaaattaaaaacaaaatgaacttttttctttatttatatatatatatatatataaacatattttttttctgtaactgctttactgagataaaattcgcatacagttcacccacttaacgtgtatgatttcatgatttttttgttgttgtctttttagggccacacccgtggcatatggaagttcccaggccaggggtcgaatcagagctacagctgccagcctacaccacagccacagcaacaccagatccaagctgcgtctgcgacctacaccacagctcatggcaacaccagatccttaacccactgagcgaggcagggatcaaacccatgtcctcgtggatactagtcaggttcattaaccactgagtcatggtcacaggaactccagtgatttttttctagtacatccacagagctgtgcaaccatcatcgcaatcaattttagaacattttcttcaccctcaaaaaaagcctttgactatcaccccctccctctcccatcctcccatccctcagtcctaggcaaccactaatccagtttttgtttccatagacttgcctcttctggacacttcatataaatggagtcaggtaatatgttatcttttgtaacttgcttcttttgctgagcgtcatgtttgcaaggtttatctgtattgtagcatgtgttaatacttcattccttttacagccaaataatattccattgcatgggtatatgtaccatgtgtttatccattcaacgggtggtgggtatttagatgggttctaccttttggctactataaataatgctactataaatatccatgtacaagattttatgcagacataagttttcctttctctcaggtgtatatcctaagagatacacgtgaaattgctgagtcaaatggtagacctctgttccactatttgagaaaaagccagactgttttccaaagtggctgcaccattggacattcccaccgagggtatatgagggttccgttttctcaaaattctagcacttcagttgtctttttgatttctggtgggtgtgatgtagtatctcattgtggttttgatttgtattccctgatgactaacaatgtggagcgtcttttcatgtacttattggccctttgtgtatcttctgtggagcaatatttcatctgaatagcaagtaataattatcccattacttattaatctaataaaattagatgtggaacatcaaggcctcaccaatgagataaactcaaagaacctctggctggggtagacatgaccagacccttatcccctaccagtctcattgcaactgttacaaagtccttgagcccccctccccccacttcagctacctggcctacttcccgctgcccctaattaagtatttggcccactcctcaccctgcccctctagggaacttatacacccccaaccaacgagcaagcgtatcttaaaacttctgctctcgcctttgtccctgggctataaacacagacagcaccccacacttggggtcggctctccctgaccatcaagaggtcatcctgctgcattagcagtgtttcttgcctcaataaactcgtctttctttttatctcgatctgagtttggaaattcttttccaacccactcacagactacaacattagattaattttatcacggagttgtaagagttgttcatatactccagaaacaagtcctttatcacatgtatgattcgcacatgttttctcccattgcacaggttacgttttcactttcttaataatgtcattggaaacacagaggttttaattttgatgaagtcggatttgttaatttttttcttcttttattgcttgtgcatttgatgtcattgaagaaaccgctgccttcttcaaggtcacaaagatttacccctatgttatccttgaagagttttatagttcttgttcttacatttaggtctttgatccattttgagtatatggtgtgagataggggtccaacttcattctttcgtgtatagatatccagttgtcccaacaccatttgtggaagagactattctttccctactattatcgaaaatttgatttaccatagaaatatgggtttattttcagcctctcagttctcttctgttaatctctatgtctatcttatgccagtgccatactgccttgattacttttgctttgtagtaagttttgaaatcaaaaagcgtaagccctcgaaacattgttcttcttttcaagattattttggccattctggggtccttgagtttcatgtgagttttatgatcagtgcatcaatttctacaaagaaatcagctaaaattctcatagggattatatcaaataaaatgaacttttaaaactgtagctattttgttattattttacaggaaataagtagcatttcactttatgtggtttcatgagactcagcttctgcgagttcataaatatcagtgtgtgtcatcatctagatccggaattgccaaattctaactatgttgcttcctacgtacctttcaaaaccatgcttccttctccatctctgttgtactcatccagttcaggcccctcagcatttccagaagtcatcttccagaagtcatctgcctttattctggtatcctctaaaatatcctccaacttgaactcaaaatcagctcactaaagcccaagcatgatcctgcctcctccttttgaaaaacacttcagtggttatccgttgtcttcaagataaagttcaagttacttagcatcctaacaaggtccttccaagtttgacttctgctcacctgctgctcatctcctatccctcgtcagtaataccaaaccatccttatatttcaggcacgtaaaaagtttctagtcccagagtgcaccaagcgcttgtatgcctccctgcagtggtgcatatgtgacctttgtctggaatgctttcccctctcatctctctggaaaacttttatttcatctttcaagacttggctcaaatgtcatttccactcttttgttagtgatgataatgacgacgatgatagaacagacattttatatcagactttaaatcataggaagtatgtatgtaaaattgatataaaaataaatcttttcctactcaacctctagtcacatcttttgtactttcaatttttactttatatattttgaggctctattattaggtgcatacgtatttatgattatcatatctttataatgtattatccattttattattatgaaatatcccattttctggtaatactcattgatttgaagactgtgttactgatatttttatagccactctagctttcctgtgcttgagtttttcatgatttatctctcctcatccattgattttcaaccaattagagtcttgaattaaaagtatgttatatataacacataattgtgctttgcatttttttcttttttcttttttggccatgcctgcagcatatggaagttcccaggccagggatcagatctgagccatagctgcgacctacaccctagctgcagcaatgccagatccttagcccactgtgccacagtgggaattccatgctttacatttttatcatttctgataacttctgccttttaattagagtgctaatccttttttatttcatatagttagatttaagcctagaattttgttatttgttttctgtttgtgccttcttttttttccttctgtttctttcctttctttggattaattgaatatgttttaaaattccctctttacttgtacctctttgccttattttttgagaaggtaatacttcagtctaaagttaccaggtacatccctaattcttcacagtctgctaagagttttgaattataccatttcacatgaaatgtagaaagtttgcaaccatgtggtcgtccatttacatctctgtcctttatgcaagttgttataatgactatacccacctacactataaatcccataatacaatgttataacttccatttaaacaatgatttgtattttaaagaaattaagaggacaaaattacctttttactcatccacctatttaccatttccagtgattttattccagtgtagatttgaggggtttttttgtttgtttgtttgttttttatttttttgtcttttttgccatttcttaggccgctcccacggcatatggagattcccaggctaggggtcgaatcggagctgtagccaccggcatgtgccagagccacagcaacactggatctgaaccatgtctgcaacctacaccacagctcacggcaatgccggatccttaacccactgagcaaggccagggatcaaacccgcaacctcatggctcctagtcggattcactaaccactgagccacaacgggaactcctgtagatttgagtttttatctgatttcattttcctcctgtctgaaggacttcctttagcatttcttgtagtgcagttctactgctgacaaatgcttacatttcctttaacctaaaaatctgtctttcacctttattttcgaaggatatattcactaaatatagaatttggagttgacagggtttctccacccccctcaacccccatattttaaagatgctgtgccattatctaatggcctcctttgtttctaaaataactcacagtgtgtccctcccactttgtttacaatgtgttattttctctgactgactactttcaagactttatttttattttgttttcagaaatttgactagcatgtctttaggtatgttgttgctgttgatttttaagatcctgcttgggagtttcctgagctttttgaacctgtaagtttatgcctttaccaaatttagccatttcttaaaactctttccccaaacattttctgtcccatttttctctttcccttctagcattccagttacatgtatgttagatattttgatattgtcccacagttccttgaagtactgctcatttctaaaaaatgtgtatgtccttcagaatggataccttctattggtctatcgtcaacttctctgagtctttcttttgtcatctttcttctgctctgaaagccacccagtgaactttttatctatgatattgaaattttcatttctgaggacttttccaagaaattttgagatttgccatttggttcttctttgtagtctctattctgctgagatttctctatgcttttatttacggcacatattttatgtgccataaaatggctaggaattcctgatctctttgttaaagggattgcaatttttttcctgtataatacatttttctaactccccacatgttagtcttattttatttccttactgagacagctaatccttatctaagcactattaaattaatgggttattttatttaattctcacaacccctctaaggggaagatggctttattatccccattttacagatgagaaaggtgagcaagagattagttgtgctaacttcttctaaatccttgtctgctattccagtattcttaatcatcttggaaatggtcatcattggcttactttttctcaggttttcttattttttggtatgttggggaattttgtatcctggacgttgtgaatgatacattgtagagtctctggattccattgtatttctctgaagaaaactggtttagattttagttggtagttaacttgactgacctcaaactgtaaactctgtctctcctctgtggttggtggcagctcaaatttaagttcaattgtttaagcctcagctggctgcttggtgtctgcccccatttatgtgcaatttaagggtcaactagagatttgggttccctctctcctttcttgggttcctcctcccctttttttagtatatgtgctgctgaagcgagcatcctttcttgggttccccccttcccactctacagtgactgtggttgccctaaactgtgtcctcttgttcatcaagccaataaaaatgtaggttttggggtcagaatttaacgccctacatgcacatgctggggtctgcctttattctaaatgcttcttaagaagaaagaaactcactctgtgatgtccttttcttccaagtgtcaacttccctccagcatctgcctgcttttgttcattccattatcttcaggtagttgatttttagatttttgtctagagtttatagctggatagcatcagattgtgttcattcattctaaggaaatttatggtcaggaaaaaatggctaggaattcctgatctctttgttaaagggattgcaatttttttcctgtataatacacttttctaattccccacatgttagtcttattttatttccttactgggacagctaatccttatctaagcactattaaattaacgggttattttatttatttctcacaacccctctaaggggaagatggctttattatccccatttacagatgagaaaggtgagcaagagattcagctgcttatcagagtaactgggcatgaacggcattccaaagggctgctccctctataacccattttctttctcagtctggttgtgagatacagcttgcacagagggcaggttttcagaaccaaggccatagctagaggcagaaggtggaaggagtcatctatgactttgatcttggcccagtccttgttagtcatccaagtcagaggtttatcctagctgggcataaccaacagtatccactctagttaacaaggatgaagtccaagagagttcaagggaatattaggaagttgacaacatctctaggagggccagagggtatggtattagggtctcgaagccacttataatgcccatggtaccctggacactggctgtctcttggctaccctccccagagtgaagccccacagcacctgcttttcatatccttcattcatgccctgactgctctcaccagcatgtgtgttgtcatatccttcctcgcacgtctcatctcactgtggaggaggctctgagaaattatttctctgagtggggagatgggatattcctggacctcagaagggtattcaaatgctcctgggcaaccagagggttcaatagataggcacattccatgtccatgagctactgggcagattccaagtgacctgctttacttagaataatttctccccataagaggctgggacaacccctttctgtgctgcctgactttgagtttccctcgtttagagaaaagccaaactccataagcagacggttttatgggagcatgactcatgctggtttttgtttaactgttattgcaccttgactataataaaatgactgcatttggaatgtaagctgacatttctagtttttataagaaaaatcagattctagagatagacttatggaaactctccatttgctcagggtcacctccaattgttgaccaggtagacctggatttgtcattgtgggcccctggttcccagaactcctgccttaggaaccaggccttggggggttatctagaccaaagggagtgtctggaaagcttagtgtgttatgtgcttttccaaatgacctttggaattttgactgaaaatggtagagacggaaccagttggaggggcgggatgggataaccctgccctgcctgcagtccctgaactgctaaatcaatatggacaggaaactcacgtccatatttgggggaaatgggctcatgtggtagaaaagagttttactctaactgactggtgtatagattttcttggggacaacaatgaaaataaagtatcatttgggcttttatggtcattcagttctgttcaatgatacaaaaagtaaactaaatccctgtcttcatctcctaaggctggtgtttgcaaggtgtattcagcagtcatacgttttgggatttttggagcaatcctaattccttattattaaattatggtttaattcttgtatatatctttaggtataatacagattctcaccatttgcaattctctagtttccctgcttatgtgccctttctgagatgttctaggcagagataacacatatgtagaccctccttctctcaacactaatggaagcacattttctgcactatgtgcgtgtgtgtgtatgtatatatatatatatgtacatattcatatacacatgtgtgcaagtgtattttatacatatgcatatatgaatgagtagggcagagctctttccgtatgtgttcatgggcagatcttcctcctttcttgtaacagagaaagaggattattgttttgttcgtttgtttgttttgtctttttgtctttttagggccgtacccgcagcatatggaggttcccaggctaggggtccaatcagagctgtagctgccacctacaccacagccacagcaacgccggatccttaacccactgagcaaggccagggatcgaacccacaacctcatggttcctagtcggattcgtttccactgtgccttgatgggaactcccgagaaaggagattattaaccagctatctcatggtggttatttacccagtcttctattaatagacaggcattctgttgtttccagtcttttgcgactataaacaatgttttgctttgtgcccacccagggacgcaaaaaaaaaaaaaaaaaaaaaaaacaacatgttttggtcactatccatatatgtacctctttgggcacgtatgagagtttatgagagtaacttcctgtaactggaattacgaggtgaaaggtgattaacactttggaattagacagccactgctaaattggcctccaaagaggcagatataatcctaacctctttgccctacccacttatcagcacggcacattatacctgtccgactgctgtcattttaatttgtgtatcttttattcagaatatatgtttttgttgatttattttttgtcttttgtctttttatggccgcacctgtggcatatggaggttcccagggaaggggttgaatcagaaccagcagctgccggcctacaccacggccacagcaacacgggatctgagctgcatcctacacaacagcggcatatggaggttcccaggctaggggtctaatcggaggtatagcctccagcctacaccaaagccacagcaacgtggaatacaagccgcgtctgctacctacaccacagctcacagcaacgccagatccttaacccactgagcaaggccagagattgaacctgcatcctcatggatcctagtcaggttcattaaccaccgagccacgacggaaactcccagaatatatgtttttaaacaaacatatttaaaatccataaattctctgattatacccattctcttttggattgggattgtctttcatatggcttcacagaagctttttatattataaattatacatataatttacagtataaatgagctttcctcatttaaagtaaagatgatttgttaaatgtagaactagttgtgattcagcgtctctgactaggtgtggttcttcatatgaacaaattcataaatcagaaaccattcctagagcacgattccagattttttttttaacttggaaaatagggccattaaaaagcattttgaagtaatttacaaaactatgcaatccatgatggcattgcacacttgcagagtaaattcaagtgactttaaaacattttctagtttatacaaccatgaaagttataaaactataaaatttaagtgacttcaaatattttgtagtttataaaattatgtatttatatatgtttaatatatactcatccattttaaaaagtccaatatacttaaatgtataaatttaaaaatacaagttctctctcttctgaccaaggcagcacccctgttaacagtttggtgtgaattcctcctaacttctttctagacaccaacgatatgtctctaaatcctttttttccctgaaaggtgggagcggcatgctgttctgcaacttgattttttttcagtgaaggattaattctggatgtcaaaaatgtaacttttgcaaagcagctttgaactctgtaagctgacagtatattgccagactacagcaggacctgatatggctgccttcgggagtgatctagccccagctaatgggctagaacagatgaagcttttggagacatatcagatagctcactgtgtgctgtcatttctcctggagctccgatgtctgcatgtggagagtgttttacacacacagtggaagggcttagacgggaggggaagatgctggttataatcacaaagaaagggagctagaatggaggccttcgtgagccagcacctaaaaactcattttgatttttgaaagattgggcgtctcaccttctttaaggatttttgtttttaagatgcaccttgaggcataaatccatacaatccaccaagagaaggaatggggtcaaaacagtgggtgcacagaagtgggctccaaggagcctcaccccatctctcaagaagtggggtggtctggtctacaccccggaacagctcctgcctccctcgtgctcctgtggaaggcaggtccactctctcgtgggtgggggagctgatgtccagttacctccaaggactaagtcttgctcttctggggcaccctcccagcagcgccttacagagagggggcgctccagggttgtcaggcagggctaagaaatttgcgctacacgaaccagcccgatacaggaggcctccactcaccaaagcattgtatcctggccgggctgcctctctccagagacaggggcccttttttccctcccaacaaaggcactttctgctcagccaagcccctgggggttgcatgtgcctgagggggacttcttccgagtcagcccagaggcactgaacagactctccaggccctggcaggctggcctggcatggcttcaggaggctccagatgggtctgccctggggagccctgacccaggggactgggtcctccccagcatccttcggggccacccttgatctgccctcctaggcttgggaacaaactgagttctgggagggtggctgggtagcagttttcttgaatagaatccattctgcgttcattcaacaaatatttgaggctctcccatacgccaaggcctgttctagctctggaaataggcagaacaggacagacaagggcccgtctttgtggagctggcaatctagtgggggaagccagacggggaacaagtcaaatgagtaaacaagaccgttttccagagagcacggtgaggaaaagaaaacgcaccacggcctgaccggcgacgggagaggtggctggtgtgccacgtgtaactgagaggaacctcttggaggtgaggctcaagtgaccggaagaaaggagtcgtaccaacatctgggaagaacattccagggagccagcagagcggagtgaggcaccctgaggctgggagcacagtggggagccgaggagggtctgagatggaagctgaggtgagggccaaactgttcagggccacgtggccaggctgagatgcctcatgtcctcttccacacgcgacgggcagtcactggagggtgaagcacctgagcgtggtccaagtacatgtttaaagggaaactcaaggagttcccgttgtggttcagctggttaaggactgtgaggatgcgggttcgattcctggccttgcccagtgggttaaggatctcgcattgccacaggctacagcctaggtggtagatatggctcggatccagcgtggatgtggctggagcatcatctggcagctgcagctcagattcgacccctggcctggggacttctatgtgccacaggtgaagccctaaaaagaaaagaggaaactcaggctgctctgtggggagcgttaggggagcacagagccaagagggcagtggagaggtagtcaggcctgccaccgtggtccaggtgagagagcgcggaacccggctgcctgatgaggtggagggaagccaggagctgggatgcagatttgggatttctgccaactgacatcctcacccagaggcagggcctggtatccctgctctcctgcttgcactgaatgggcgctggccccgccagggtgctgagaccctgcctggcggatctcccatatcatctttcaactcatgtaccaggacccctgagctggacccccacattagagggccccactcttgctcttctctggctgcccccttccctccgggtgacgaccctacaggcaaaggaacttcctgccccaacagcctagagccagctgcctgggcctacaagggccgctctgccaagcacttggtatcaaacacaaccctaggtcaaagggtgatgggaaggggcaggtttcagagattatgggaaatatcagagccttgtactgggcatccacacccacctggctaagggcccttctctgtgtgtgtgggggggggggaatgacacaaggggttggaagagaagggggtggctgcctctcctggtccttcatgttccagggtcaagttcctttttattttttaaattttttttttgctttttagggctgtacccacagcatattgaagttcccaggctaggggtctaattggagctgaagctgccagcctacaccacagccatggcaatgccagatccgagccgggtctgcgacctacaccacagctcatgtcaacgccagatccttaacccactgagtgaggccaggggtcgaaacctcatcctcatggatcctctcaggttcgttaactgctgagccacgatgggaactccgagtcagattctaaatttacatctggcctctaggacatgacaaagatttatttgtccaggcaggaaggtagaacatattttatctaacagtttcttcatttgattgataacttttaatgtttaaacagatggtatgggggctgccatttgtaatcttgtcctggatcacacaaaaatgtgggctgggtccaagcccacccaacatccacagcttgctttctgcagtgttccccaaatgctgaaggagcagttctgggatccattatctaccaaaggagttttctaccaagatagaataaaagacttttcctaagctgctaaacaaatttaagataatggatattctttttctaaaatctttgctgcccgctactgcttatctgctagcagttgcaagcacataaacaggaaacataacaaaagaaaccacacacatattggaatagatgaaaattgagggaaaactggaaacagctgagtctgacagtgtggattttttatggactgaagttctcatggttgtgatttcatagggtgtacattcaatatagtcatgtttcctaacagttaaatcatttctagcacacagtgacagaattaatgatgccttgtaatattctgtgatttctaggtgctttgtcctagaacaagctgagaatgccctgtaatgagctattgcccccaatttacagaaaagaaaacagaggctcagagaaattcaataactttcccaaggtgaccctaatacacaaagtaggggttttaatgcatctgaggaatccctgcactaccagaactcccagtgagacagagtaggagacagggtaacaagaatgtcttctcaggctgctctcattgtggtgccgtggaaacaaatctggctagtatccatgaggatgcaggttcgttccctggcctcagggacgcagcattgccgtgagctgtggtgtaggtcgcagatgagtcttggatcccgccttgctgtggctgtggcataagccagcagctgtagctccaatttgacccctagcaaaaaaaaaaaaaaagtaacttctgattgttttgcttgtctacgttttaggaaggtcgttgagaatattttagggaatcactgtagtagacagactgtattagacactcatgccccctggtcagagcacaaaccatgcctgaaaattacagttcctgtcttttatcttaattagaccttattaattatcttaactagttctgctttgaccaactctacatccacgtagctgtcatcaaagttagaaactaaatcagaatgatttgtcctctttgaacttgctcttcagcattcgcttgattgtcaaacttctcttatatgcattgccttaggaaaggcgaatcgaagtaaaataaaaacttccacaggctctgcctatcttctcaaaatggtcagcgtggtgaacggctctgggaccaggctccatccaggagcacccaaacctcgggaccacgattctcagagcgggaggctcactccccccccctacacacccctcgcagcctgccctcccccccagctcggcatcttcctctcccgagctatccggagcataattcacacctatgaaatgcattttcacttctgttgctgattttctcctttgtaaaaagtagagccagagcacgcctcttctctcgccgggcaccccgtcagagcccctgggtccctgccccacgcccccctgctgagtacattgaggccaaggcttatcagaaagctctccctcttctctctcctcgaggactgccaagtgccatcaatccaggcaacgcagaaggatcgtaaaggtgttcggggaagatttatgagcttctcttattactggctctgtctcaacagagcgtgagccacagagccatctccctcccaagccatcccagcaaaatgctggctgcccacgtgatttttcaggccatagcccccttccagccatgggcacttcccatggagagtggctctgccaagcctggggtctccagccaattcttgcttctggggttctgcagccaattcttgcttcacagtgtcgccagccccaggacgctccctcatctctgggcagcccacccacaaacccactacttggcctctaaacagctttctcctcatcccaagacacatgttctcttagacttcaaaggaaaaactagattatggaaggcagatcttggcgactgttcctgggtcatggtcttctcttccagaactgcaaatcaggcaaggagtgtcctatgggtggtgagaccccacgacctccaggggcttgaacgagaccctctattttccctgcatcgcttatgttgtggacagacagtaaccccagccggaagccaggcattcagcccctacccagactcccaagtcctgacaatttgttcaaatccatccctgccctctgtcccccatcctggggcctcaggttgccactgcttctcatccggtcatcccttccctctccagcccctcccactgtggcctttcaggaacccacattggatcacagagctcccctgctagaagctgggcggcaaaaccctgtgagagagtcctccttcacccggagccaccttggccgtgagcccagccctgtctgtgcatccagccttatctaggcccctccccagcaatccctgcactccagcccatcaaacatcttgtcctttgttaccagaacacgccatgcactcgcagccgctccacctttgcacctgccattctgcaaattgcacctctccttctagcaaattcctgctcatccttcaaggtgcggatcaaaggtccacggctgcatgaagccctcctcaacctctggtctcagtcagtgcctgctgccctaacaaaataccagacagggggcttgaaccacagaaatgcattttctcacagttctgaagcctagaagtctgagattagggcgccagcattgttgggttctgctgtgggttctcttcctggtttgcaaatgccagcaccttctccctctggcttcacatggctgagggggggggggcgagagagcccatcgatcctatcagattagaaccccacccttatgatcttatttaatcttaatcacctcctgaaaaatccatctccaaatagagttacaatgggggttagggctcaacatatgaattttcaggaggacaaaatttagcccatagcactatccccccaaaagctggttgcaccctcctccaaagccccatatgtgtctgcacagctttattatagcacatccatcgttcatctgcactggtacctcctaaaggacatatgacgaattgctgcactccatgcttctagccagtctcgtttctggctctttcggcatcacctcctcattgaggaggcccttgtgactttgtgtgaagatctgtctgtctcccagctatgcccaaggctccgagagagggcaggggctgcttctcactcacctctgaccactgaacaccttgcacagagctggcgtagtggggagctttctggttctgctgtggaaggatctacagccctggctgtggtgcctctcttccccccacaataaggctttattgataaggccttattctggtactcccccagtcctgggggcctctgcaatatccccagatcctaagaagagacattcctgatacctgcacaggagagcaggtgtatgctaaaacagggcacttccaattgtcttttctttgttcctgcactagggcagaccgttatttttagcattgcagaaaaaatgaaaatcccatgccctgttttggggtttccctccagatctccacaggtgcacgtgtacaaatggtcctagatccaggcccagagaccagctcctcagctcacataggttattcgtttaatgtatctactttcactcccagtccaaaaacatcgaactgaaaatatcttatgtatgagctctgggccaaaaaaaaaaaaaaaaaaaaaaaaaatgaagcagttgggccaataatcactgggaatatgttgaataggaacttgattaaagcagagtatgtgttccccagacgaagttgctgctttactgcgaccacagttggaacctccgcctccccaggccttgctttcgcgctgcgcctccttgagggctcctgggcgggctgtggcaacttgccagcctttggaaggccactgtccgggagagggtcctgacccgcacagcctggcgattttcaaacaggcatacttccaaaacagcacccatggcggcgatgggcctcagcgcacttaatctaaggggcgatgagggcgggggaggtcgaggcggggagacttgctggcccacgacgcctcaagggagccggctgtccccgcgatcccaaagaagaacgcgcgaggcgccacgtggctggggaaaggcgctgccataaaccccttccccctcagctgtgtgtgtgtcggggtggggggggaaggcggcgctcaggggtcacccgggccgggccgctttgttctccaggattctggggactcagcgccctgcaaaccactctgcagggtttcccagtcggtgttcggactctaggccaccgagactccgaggcctcggctccctcgatattctcgctccggtactggagcccacctgaatggggaggagaggaaaaggctgtgggttccaaccccgctgaccgccaaccccgcttcgcgatccgcgcccctgggtcaaattcgcctactccctctggtgcgtcccccctcgccccgtcccccagccccggaatccagctcggccagtcccacaggaaaaagttggcggccccgcgccaggtagcagcgccaactctgcgcgcctagcaacacctagaactccggttaggagaagtaattaaaaagttttccagcccaggcggatggaccgaccgggtcctccaaactccctggactcgtccgcctccccctccctccactctccgccgggtcctagcgccggtcggtgcgaagccgcggaaccttggagtacggcaagaggtggggctggggagaggtacttctgcgcgaaatccgtggggacgcctggagatcaaaaagacgcaagatccccgcgctccggacctctctcctcggcctctccaccgggcagtgtacgctgggccgcaggtccaaaactgttccctgcccccgaacccccggtgctttctctacgccgccgctttccagagagcctggacccggacaaaactggttctagtttttaaagcgagttcgcgccacagaaactcagagaccaagacttgccctccaccaccaacagctctctgagcggcgtctgggtgctcccgggctcccctgccgttggcagacggtgggtgccctggggaagccagaggagagggcctcaccagggaccacgtgtgcgccggggcaaccactcctcggagggtggggcaccactcgcaggtcacccttcagcttgctgcgcagggggtagtgggcgttgcgccgccgcccctcccagtcagtgctgcgctcctgccccactcaccgccctctaacgatcgcaggcgctaagacccaggaggcgccgctccaccgtcgccgaaggtccgagaccggtttg**gacac**ta**gaggg**cgatggccgccgcggtataaataccgggcccgcgcaggcctggCCATTCGCGACCGGGAGCTGCGCGGGCGCGAGTGAGTAGGGTCTCCGGGTGGGCAGCGGCAGCAGCGGCGCCCCGCGCGGGCTGGAGGCCGCCGAGGCTCGCCATGCCGGGAGGACTCTAGCTCCCCCATGGAGTCGGCCGACTTCTACGAGGCGGAGCCGCGGCCCCCGATGAGCAGCC…

Supplementary Figure 3

Putative EBF1, SREBF1, C/EBPα, and GATA-2 binding sites in the porcine *C/EBPα* gene promoter region

TFSEARCH searching transcription factor binding sites (ver. 1.3) was used to identify SREBF1, C/EBPα, and GATA-2 binding sites in the 20-kb upstream region of the transcription initiation site of the porcine *C/EBPα* gene. The predicted EBF1 recognition site reported by Jimenez [26] is enclosed in a box**.** The putative SREBF1, C/EBPα, and GATA-2 binding sites are indicated by magenta, yellow, and blue highlighting, respectively. The first exon of porcine *C/EBPα* is capitalized, and the coding sequence is underlined. The wavy line shows overlapping GATA-2 sites. In total, 1 SREBF1, 3 C/EBPα, and 24 GATA-2 sites were identified.
