# Supplementary material for: MicroRNA-33b downregulates the differentiation and development of porcine preadipocytes
Source: Mol Biol Rep. 2014 Jan 8;41(2):1081–90. doi: 10.1007/s11033-013-2954-z (PMC3929038; doi:10.1007/s11033-013-2954-z)
Supplement: Supplementary file 5 — Supplementary material 5 (DOCX 25 kb) [file 11033_2013_2954_MOESM5_ESM.docx]

gatcctgtgttgctatggctgtggtataggcctcagctacagctccaattcaaccccaggcctgggaacttccatatgctgcaggtgcccccccccccaaaaaaagtactcaggaaaacaaacacccactttcttagaatggtttaattttagaccatctctttctcaaaataggatctatagattcttggggagccataaataaattcttagagagtttcagaattttaaaatactgtgttgttatgtcagtggtaacctaaaaggtcattagaaagcccagatttcacaattcaaggacattttcatggtttcttccattagatttcttgacctgggatagaggacctctggggagcttgtgaatatagccttggcaggtctgtacatttttttcttcctttgaaagagacacacacattagtgatgcttgagcaactctggtcaaatgcagctcccttccttttacaaaagagccctggagaagttcccgttgtggctcagtgggttaagaactcgacatagtgtctgtgaggatgcaggtttgatccctggccttgctcagtggattaaggatctagagttgccccaagctgcggcatagtttgcagatgcagctggagtccagtgttgctgtggctatgatgtaggctgcagctgcagctctgattcaaccccgagccaggaacttccaaatgcagcaagtgtggcagtaaaaggaaaaaaagaaaagaacaaaaggaggtctggagaaattaaatgatttcctagaataacacagcaagttactgaaaccaaccaacactaaaactcaagtcttccaaggctttcatctatgtttgttgggctagtatgggggtgctgcagggatcatggctaccttgggtttagaatgaaatgggaaaagcagacaatgagaaagcaatgacaaatgatgagtcctatgagggagggaaagagtaaggagctgtttggaataataacagggtgattttacctgatctgaggatttgaggcagtgatttttatgctgacaatcaaactgaggcatcagctgagatatttcaatagacacatcaaacaggtatttgtatgtagggctgaggcacagttaagaccacggggacagagatagagatttggtctcaatagatccttcatctttgaagccttctgtaatgagattgcagagggagagcatctaggattggaagagtgaagagcctagaaatagagaaagaaccaaccttcttagtaggataactggtatcgaagaagccagaaagaagaggcatgatgaaagaaaggtcaggacccaggggtctgctctctgatggaaaagtcacattgttcttttcagcaaaatatctacctaccttacatgctgagactgttttatgctaattgttgatttaacatttttccaactcttttgccagagggtagtcttgtgaaatgttgatgaactgggctgtacattgttcttggtaaattgagtttctggttcagttggtcagtcagaaagcccttttcactcctggttatgtaaaatctttcttgaacatagtaagccatttttctgtcttggtaaatattgtgggattaaaaaaggtttaaattgaatttagataattttgagttgatgattcaggaacttttaatgtctttgttgcatttttgaatagcaaatgacattttgctaaagatagagaaaatattaaaaatcgagaaaatattaaagatcatctcaggaaatgctctggaagccactttaaaaaacaaatcactttttaaatgcccttttttttttgcttagtattcatttttttttttttctaaaggtataggaacaacttttagtacattaagggcttttgatggttgatggaagttatattgcatttatttttatttgtatgtggggtttttatataattctaacctcggagaaccaagtatatcagtacattcctcagcattgctaatcatcaaaatatgtgctcttccataactgacctccatatgtaatgcagctatataaaagttattcctactgacgttatagatatacatgtgtgggcagtagagagcaatatcaaaagttctatctatatcagattcgagtaaattaagtcatgaggcacctgtacagaggatctttccagtggtgataatgctaaagaaaaatcttttaataatttaaagcaccttttaaaatgtgttctgggaagttcccttgtgacacagtgggttaaggatccatcgctgtcacttcaggggcttgggttgctgctatgtggggcatgagtttgatccctggcctgggaatgtcctcatgctgcagtagtggccccaaaataaaataaaataaaatgtggtctgggagaaggaaactaatacttacactcactctgtgccaagcagttctaagtgctttgcctacattatttctttcaatcctcacaacagttctatttcacaagtgaggccactgagacttaaagaggggaagtactttgcccaaagttataccccagtacaatgtggtgatactcagactctggttgaagtttatctcactttaaagtaaatattctttccatgtacacctttcttcctccctggaagtcaggggaccgaaatgttatagctcctgctacatcctctaaccatgtaacgctggataagccactactagcctcctgtaagcatgctaatatttgcatttgctatcccccagacatactttgatccttcccccttgcctagagtgctccatacctcctctccaggtgttcgttcatgcattgttaatccattcatcagttggttgggcaacagtagtctttattcatacaatgatacctgttgaggttctgtgacacagatatgaacagatgtatccatgggtcaggggccataggtacatggttgctgttttttaggaggccatgctcttgttgtatctatatcttgctcatgtataggccatagctgaaatcccatatcttccaggaaccatcccatcttgataatgctctcctcgctccctgtgaaaccgagtcatttttccatgtatttggcaattcatcaggtgctaccttgtgacatctcttctatcattggctggaattcttattcacatcatgaatcattactcaacttttcacctgtttgcagcttgtttcttcagcaagatataaattccttgagggcaggcttactcttctctgtatttcccacagtgtcttgtaaatggcagtgctttttgactgggttcattcagtgaatgtttgttgatttgggttaaattttttaagttcagattttacttcctggtcgactgaccttgaccttagcttctctgggcctcagttttcaataaaatggaaataattcctatttctcaagattgaaaagataatgtatgtaaaaatgctttgtgcacttgaaaagactgtataagccttacagattatttgatactttgttgtggaaatctccctatccttcattcctccaacagctccttcatactcccaccatttccttcaactgttcacttgacctaattttgtctctctggttctaccatataactctttgcagtttaacatctttatttgctctaaagatacttctaaatgttttccacctttattttgaatagccccaaatcttagtcttattcatgactgtggatgtaaatgaacttatctgatccatcagataaaggggccccttgtgatgcaaaattggagtcatagtattgttaatttttttcaaggtagcttcatctgttcacttgcagcttcagattgatgtctagggtttgttgttaggaatttgtaattatggtttttaatctatattagaactgggtatactcttcaaagactatgttatgactggtaccatccaaggctatgttgctttagggaccgtggcaaatttagtaataacccccaatacagtaatttcaatagtttaggtttaggattaagacttcccaagtgttaagaatatactgcactgagttattatggtctttgctattttcagtgctttggtgagttatagccatcaaaccaagctttattttactgtcctcactgaagtaagcttttctagaatgtggttcttatgtgggtaatgtttgcccacctacagaaaaccttaaaataaatatattgactattgttatttaggttgaaagatattaaaatgagatttgtaatccaaatgggcattacttacttcacatctgtttccttgaaaagtggccaaatgcgacaattcttaatgcaagacagcctggggtaaaacccttgcaattcccaccctcctttgcaaaacaactgccctgtgcaaacctttaacattacaaaaaagaagaaaagagaaacaaagaaaatttacgttaattatagattttcaacagaagtgctaatgtaattcccagtttaagagctagcaacccttacaagtggaagatagtgtaggaaaactatggcagccttgacctagtttctggttgccaggaccaaaaacaccgtcaaacctctctcttttaattcccctcattttgctttcttttaaagtgattctgtgtcctggttgcctgtggcagtctcagtttatgcctgtggctttctttccctcttcacagtacccagtttgggtcataaagtatatggtcatgccatttaatccctcagagacagtgctggggaagcgtaaagagtaggaatgttgagaacagattaaagggaagatgatcttcaggttgagacactgctggaatcagaaagctactgatgagagttctcaggaaggaacgagagctgagctagaaaattgatttgtgaacacattttgaacatcacaagatccaccagattaatgctggttgctcttggggagagtggagaggcggaggacaagaaccaggtcaggagcttgtggtatcttttatgcaacagtaacgttgggcttcctgtctgaggtttgccatgttgatacctacctcttgatactctatgaggactgtgatgctggtatctagtagaaacacttcagagaaaaaaaaaaagatggaagataatagttctttagaaaaatggaaacacattattcttgataactttcatcttctgtgaaaggcttagctaaacatctctgctatttaccttaataacctacttagaaatttaattttttcaactattaaaatgcttgcttttttgatgctctcttttaaagagcccgctggatttgatagccatttttttaaatgctcaattgttgcatggtgtctttattttacttaaaataacttttcaaattctaagagtaatagctgctattgaaaaagatgtagaaaagtataaagcaaaaaataagcactcataatcccatcacccagaaatacctaaccgtagcaacatagcatagttcctttcaactttctttcacatatatattttttaatgtttattcagtgaagctcctaaattataaccttatggaaaatgcttctagtgttctggtaacaacaggaaaataataatatcttacttttgcatagagctttaaaatgtataaaattcttccatatagtaacatatttaattctaatatatatttaattccttataaggcaaggcagatattacttcccattttacatatgagaaaactgaagcctggaaatgctcagtgatttgtccaaagacatagtaagttaagtagtagagccttgactagaaccctaggctccttcactgtccaagactttctatgtacaacttatgaaattaattatactagagcttttggtaccccaaaacagaattgagtctaaagaactaatgatgaaaaccagggaaagaaaggaaggaaaaggagtattgtgtttgaataatgatctttggggttgcagagggtcaggcagaattcaaagaatgataatcatcttttttactttcttctgaaagtcaaacaaggggccaagcttcttgggaacctttaagtttggagtagggcactttcctaaaggcctttgaagacaatacactacctgtttggaattaacagtcaatcgagtcatgaacctctaatgctatatattttttctaatttttattttattttatcttattttgctttttagggccacacctgtggcatatggaggttcccaggccaggggtcaaatcggagctacaactgccagcctacaccacaaccacagcaacaaaggatctgagccgcatctgagacctacaccacagctcacggcaacaccggatccttgacccactgagcgaggccagggattgaacctgcaactttatgcttcccagttggattcggttccattgcgccacgacaggaactcctgtaatgctatattaagaataaatgaaataaaaggcatacactctcatgttttcaaaggacttaacaatatacctagagaaagaaaacaaacatgtgaaacaagtaggtgtaaattatgtggtattgactgtgtacagtgagggtaagaactaataaattggcattcaaagagcatttactttgtgcccatcaagaaaagtagtatcattaagtgttacagcataaataagatcaggaaaccagaaacaagtcctggtggatggcagaaagtaggaatatacacattcattcacatgctaatgaatccgcttagcattttttgagcagctacagtataccacaggtatacaggggtgagtagatacatccttttgaatagaactgaagctatgcgttaggaaatagtgaacagtaaggtgagtgagtaagtggatgtgtgggcagctgacagccacaatactcttaacctgttaataatatgttaggcatgattgtcactggggactagagtttgtaatcagtgagctcttaacctataaaatttattgaatgggagttcctgtcgtggcgcagcagaaatgaatcagattagaaactatgaggttgtgggttcaatccctgtcctcgctcagtgggttaaggatctggcgttgccatgagctgtggcgtaggccagtggctacagctccgattagacccctagcctgggaacctccacatgccaagggagcagcccaagaaatggcaaaaagacaaaaaaaaaaaaaaaaaagaagaagctcttataacgcttttcttctgggacttcttaagactatacatgtgtagatatccctccgcaaagtaagggactggttgaaaataatctggtttagtggaactagagagagggtgcagtaatcaagatatcatggattctagctttgactttgtcactaactctgtattatcttaggccagtcacttaacccccttgtgccttggtgtccccaactgtaaatgaagagggtttagacaaaatgtcctttcagactacctacaaaggtgaaaatctcgtgttcacatttctgttatctttgaagcccatatcccttctcaatcccctattaatccccccttccaattgctcttttcaaaccccctttcatcaccgcgcatattccttgtctcagtaactcatggtcactgaaacttcctgacctcaatacataggtctttcaggtacgccatttctaaaatgtttatctgaaagttccataggagactaaatgtcacatcattcgttaatctccctggggctatgttctcatcccctgtaactctcttatggaagctctccacagataacaatacacttccccttcaggcagtacatgaaggaaacaatggaatcatcctataaaattacacagaatgaggatctggcccattgttttctcctgctgcctaaggaagaagttgtactctttggatccccataaagattttggcttgtctacctcctcagattccaagtcacaattaagcaatctggcctccttcccacttctcatttcaaagtgtaggatacttgtcagcttgcccagaatgactccaggcattttgagctattcatctgagtgtgtgcaatagataaatacaataaaaacataattaggaggaagactgacctaaaactcaattttgtttttatttttattgaggtcgggcacacttatcttagaggtgaaaaagttgtttcttatctgtcttcatggtttggtctttctctccttccttttgtctgcaattagatgttcccaaatatttaaaatgaaaagtttattaaaatggagctcatgtttgtaaatataagaacatagcatcttaacttcctagagtttggagtgtctgaaggaaaagcatccctggagtataaagagaaaactaggtggttggaatgtggtttaattagtccacaaacctgacaggaatcttaggtacccttcctaaagctatgctgacacacagcataactttggaggaaccagttaaatttgtattctacttattccccataagttaggaatagccacttgctaaaaaactgaaagtcgctattaatggagattgttaagtaaaaaggaagccttagttatattcttaggggttaataagtggctttgtatatacaataaaaagaaaaaggattagataactaaagaaccaggggatggatgccttggttttaactgtcatggtaccaaagataacagaaaagtgaagctgacataaaattctctaaaaataattgaaaaaacttaagttttatctaagttaaacaacttagacatgtaagtattttatgctatgagccttcagtgaacagaagaagcagatatgcctaaccaagaagttttgaaattgaagctatctttctagtagcagagaacagattaaacatttatttttcctttgcagtaggaactgtagggtatctacattgctttagttcttaaggaaagctctacctcatagaaaaatgccagctaataaatgtagaaggagtgatagaatgagaaaaaaaacttcatttaatacactcagagtattgattcaggaaaagatgattgtgttaaaacctttaagtgaatgatcggtgaaactgacatgcctgtaattccacagtaatggctcagagattacttcccagttgcaagaatagaagagtaaccccacaatggagagatcagactttcatcattctggagttcctgttgtggcatagcggaaacgaatccaactaggaaccatgaggttgcgggttccatccctggcctcctcgctcagtgggttaaggatccagcgttgccatcaactgtggtgtaggtcgcagacgtggctgggagcctgtgttgctgtgacataggccggaagctatagctacaatttgacccctagactgggaacctccatatgccatggatgtagccctaaaaagcaaaaaagaaaagaaaagaaaaagacttttatcattctaagttagtaatcaatcccagcatcgctaatggtcagtcatgtctcatgtctctgatgtggtggagtaggaaacagagacatccagtctgtgggaaacaagctgaatgataccacaggaaaacaaccagacaactccagagggtgagacattctacctggtaactggcctgacctcttcaattagtctatggccttaaaagttaagtgaagctagggatggggaaggatggctgtactagactttaaaagacttaagggacatagaaccaaatggatatcttcatcctagatattagattccagtttggaacaaaccagtttcaaagaatattttgggtccactggggaaatttaaacatggactgggtattgtttgaattaaaaatttactgaaaaggaaagatagagattactgactttgaaaagtaggttaccaccagcaagtactgtctcatttccactaaaaaatacaaatactatgtgtatgggtacatattttgtatgcttatatatatacttaggatcacacacatacccagaaaaagatccagttagatatacactagaattcaacactgacaatctctgagtagtagaaatataatggttttatttttaccaacctgtgttttaaaattttcagtaatgaacatggattggttctgtaaaaataaaatgttactttcaaaaaagtttctatggatgacctgacccccttgctgtacagtgggaaaataaaataaaatagtaaaaaaaaaagtttccatgtagaaaagcaagtgttttctgtcctttaaggaaaatcaaaacgtttccattgaggaggaaaggtgcaaagactagaaaacagtatctttcaaaaacatttggcagtgggttttgtctataaagttttatctatttgtctttagtcaacatggtgtagagtcaaacagacgtgttagaattcaaactctgtcactttacaagcatttttagtttgggcatgtcactttatctctcggagcctctgttttctcatctaaaagcatagttgtgagaattaaagatgatttaaatggaagttctcattgtggtataatgtgatcagtggcatttctgcagtgccaggacgcaggttccatccctgacccagcacagtgggctaaagactctggcattgctgcagcagcttaggtcacaactgtggcttggatccaggaggtagccaaaaatgaaaaataaattaattaattaaaaaggttaatttaaataaatcagtgcctggagcagaagaggctttcaaaactatagtattatgaattcttatcaaaaatttaagtattgcacatgatgacttcattagtgaaacagtgataacagtcatcagctgaccactctgttgctcttaaatgttttatgaatgtatgtttcacaaagaaatggatacaagtacaaaatcttcaaaagcaagtatgaattttcaaagttcacttagcataagtagacccattttattcatgatagcagttagagagttggtagaaaaataagggtaactttatagccaaaaccattatcagattccatattaaaggtctattcaatagctgctgtcattttcttgttttgctttaaaggatctcccagcatcattgtgagtttcaaaagagatcaaatcactgtacaattctaggtactttttaaaaattagagtatgattacctcagagcagaaattttcacacagcaatgctgtaattttcattagtggaactatgtcaatagagaggacagcagttagtaccagagaacttcagaatgctaggatgactcatgaggaatctgtgcgttgaggcaaaaggacatttgtcagcaaaatgggctcctgaattgattgacccgatatcaccaaatgttaattatcaagttgttctggagtgcttcctttgtagaaagcactgtctctagactgtgattaactggcattcccacagagaatcacctacataaagtgagtcatatggtatgaatgctttcatgttgatagatttctggcatggttgttggcattaagactaattgattggagcaatttatttgaatgctttattgcatcattttattgagatggagcacagttggagctttggaccaggaatgatgaaacctgctttctagctcaaactctgcttctaaatatctgagcgataccttgggcaaattccttgatttctcaggaatccattttccttatgaagaattaaaagagtttgaatatattcatcatcctggtattaagttacgtgagtctgtggtctgaaaagtccacatatttcctttaggacagtttgcaaaactggtaaagtacttattttacacaaagtgaacttgcatatagctctgcagctctgagcttgctttaggattgtgaagttctagcatgggtcaccagagataggctttgttacccagagaatctccatatagttgatcttgaaaaattaattgataacgtctgatcaatgaagaagataacatgttttagaagtttaggatttcccacaacacaaatgtgggtcagttgataatactgcccatggtattttattgggctggaaaccaaattactggagaatttcaaatattacatgcaaacacaagttaacatagtttaggtggtatgtctttggctcaaaggtatgatgacttgaaaagtgtttagctgatccctagaaagtggttagacaggttaggttttttttttcttcaggtatttcaggcaagttaactttttggtgggggaagaaggaagtttccagataaaggtgcttttcatgctgtcacccaattagccatcaggtatgaaaagaatagtaactcaggattagaccatgactaagactacaatatacagaatttccattatactttaaataaacttgttgcaggcatgctgctaactactccacattagaacccaaaaggacttcaaggattattctgtcttgtggaaaggtcttctcattctagagatgagtattgtgagcccaaagaattgacacaagtctgaacccatataagaacctaaaacctaggctttctggcttcaatgttactgtgcttttccatatactacattgctccagggtcaaaaagaccttttatttatttatttatttgtcttttttttttaattttagggctacacccatggcatatggaggttcccaggctaggggtcaaatgggagtggtagctaccagtctgtgccacagccactgcaacatgggttccaagccacatccgcaacctacaccaccagcttacagcgatgccagatccttaacccactgagtgaggccccggatcaaacctgtgtcctcatggatgctaatcagatttgtttccactgagccaagacaggaactcccaagaagaccttttattttttaattaaagtactgatcatttacaatgttgtgccaatctctgctgtacagcaaagtgactcagttatatatattctttttttttttttggctttttttttgccatttcttgggccgctcccgcagcatatggaggttcccaggctagaggggcctaatcagagctgtagctgccagcctacaccagagccacagcaacgtgggatccgagccgcatctgcaacctacaccacagctcacggcaacgccagatccttaacccactgagcaaggccagggatcgaaccctcaacctcctggttcctagtcagattcgttaaccactgcgccacgatgggaactcctatatattcttttataaatatatatatatatatttatatattctttcccatcatggtctgtcccaggagattggatagagttccctgtgctgtacagtaggaccttgctgtttatccattctaaatgtaatagtaagaagtccctttaaaatattgaatacacatctactggagctattgtctataagctatcattttactgagttatttttcttttaatcaaaatgatatatttaaaatattaatcctgtatagaaaatgtttggttttttttttaatctgaaccaaattacagtttttgattaaagaagactggatcttgccttcagaaaaaaaaatgacccagatgatttcaatgacagccttcctttgtctctttccatgggaatcccgggcttgctgggacagttgctatgaaattgacagaattatttgttcttctaaaaggcatgtgtcccactggagcatcatttctcccacgttgtgttccctgtgactatggagaaactccgaagtgatgcaaccccactagaaggtggggttccgagccaacttgagctgcactaggaagaggcggcagcagagctgggcaggggggaggggagcttcagtctttctgtttgttggcagttatgtggcttcctttagagaaccttggggaaatttcagtcctgatggaaccagaagaaatttgaggctgttgtcagctcctgaggtctctgaactcctcctagaatgacccacatgagacagatgtgccaggcagctgtttgtctcatggctgtcatctccagcacaaacaataaacacgtgctttctgccaactagggacaccgtgcaaggccccagggatcctcacagatattagacctcatactctcagggagcttcctgaactttaggaactagtataggtgtcatagcacttgtagcaggaaaggaagcaaccttttaaatggggctgtcgccaaagaaaagaatactatatgtgcttttccaacttttaccaacatagcttgttttacagaaaagaaaaagcaattaaagaagttgtaaaaatcccaattcaggagttcttgatatggctcagtgggttaagaatccaactagtatccatgaggatgcagttttgatccctggcctcgctcagtgggttaaggatccagcgttgccgcatgcagcagcataggtcacagatgcagctcagatctggcattgctgtggctgtggttgtaggcctgcagctgcagctcctattcagcctctagcctggaaacttccatatgctccaggggtagccataaaaagacaagaaaactcaaattcagtccagagaatgaaatctgaatttaacctatagttctcccccaccagaaaaaaaccaaggtttttacatagattcagagaacagattgatggttatcagaaggaaagggggtcagaggggagggcaaaatggacaaaagtggtcaattgtttgataatggagggaaattagacttttgatggtgagcacactgtcgtgtagacagaagatgaaatgtaatgttgtacatgtgaaatttacataatgttataactggtgtgaccttggtttttaaaaaaagagcaagattttaattggttttcaaggataacatgacaggaaaaatattctattttataggaatatttgatataaatcattctaaacactgaatcatgctttcttaataaaacttcatacagctataacatgattttattaattacatgtttttagacattcttttactttttgttatggaatcagtcacagctgttactattctagttgccttctcctaaatataccaaaatttggggagtttttttggctgagtaatattcagattcttttggccgaaggcaagtttcatgaataatattgaaaaccgaatactggagtagccttctcaaatgccacactgacctttttttttcaaatgtcctaaactaggatagaattaagctgtcaccctcctcattctatcattattcctgattgtccccattctttctcctgctgctggtgtgcactgtacccatagtcacatctataccttggtgtctccaaagtccaagggcggacaatcttcatctcaccttacattgcacaaagcctggcatccctcagtaggggaaactgggttagcttttgtgaaagtacataaggaaggtggtctttgcccctttctcctcacgctgttaaaacttccaaggactgtctttctgagggggttctccaaaggatagaagataatagacttttttattgaaggatcctttacatgcaaagtgcagataaccagtttttgtacacaacacgcagacgtggaccaaagctccaacaccttacattacagttccattcactagcatgctctttacgcatcttgttataatatccttcaggttttgatattataaagagtaaggcactgggcaatcttaagaccttggggataaataaattcatgtgcatgttaaaactctcataaattttctggcgatcacatactgcatccactgatggcaatgactctacttaagcaaatatattatttctggtatcagcacatgctaactgaccttcagagttaagtaagccaaaagatccctcttttaggtgtgtttggtaatttttgataaatatagttaaatgtgaactaaatgagaatgggaatgttggcttgtgaatgtattatctctgatgcctagaataatacctggcactcaggaggtattggatgaagggatgcttgggtgcttagtggtaaaataaaatatagtgtcttttcaacatcttaagacaaaacacttacataattatatacaaatgagattggctcagtttattttgtccattttaatttgtgagcaagttatttcttaagtgacctagtgctacagtgtgataggacacaatattaccactgatataaagaatatgatttggagcccctttgtggtgcaacggcttaagaatccagccttgtcaccaagggctagggttagggttagggtcaggttattgcaatggcacaagtttgatccctaccctgctctgggaattttcacatgccgtgggtgaggccaaaaaagaaaaaaagaaagaaatatgatttaacaatttatattgtataagtaacgtgggatgctttgtgtatggcttgatatgttgttaagagtttaaacatttattgagcctgctaggtgccaggtttTATGTGTTAAAACtgtaatttttgttcattggctgtttttaaactccgcaaggtaagaatacatccacataatatcatcaagtcagcgcctagctccatacttgccattatagtgtgatagattaccaatagatgtttgttgagcaagtaaatgaatgaaatacaatgactaggagaggtttttgccttcaagcaatttggtctaggggaagaagggttgacagattgaggtaagtactgtaagagtcaatggattcaggcgctatggaataattagctgttaaaaataattttacttttgaatagtggatgcataccatcagaactcttagagtcttgcaaggcctagtgacacagcagatgggcaagttgactgatgtctgagagaaaaaaagaagagaatataagtcacagcaggtgaagagtccctgagtccctttgaatgaatccgtgtatagcccaaataataaaatagacaatactacagaacttgagaaccgggtgatactgccggcctcagaaacaagactgagaaaatcctcagaattatgtggcatgaacaagcgccatcttcactgaggaaatgtgggaatgagaggaagaagggcattgagaaacttgcccccgcccccaacctggccaatcctgaagatactgataaatgaatagcacagtgactcattgatattccttgaaagagttaatgtattttccctcaacttaccctgaactctatcctgatgtatgtgatgcctgatttattaaatgaggtgacttagaggagttgctgctgtggctcagtagattaaggatccagtgttgctgcaactgcaactcagattcatccctgtcccaggaactttggtattctgcggggggtggagggggtgatttggagacttctaggaataggagttggtgaaggccagtggtggactggcctgagaattaggcctggtggaaacccagattcaagttctagctctgcgttttctcatcttattaatagagtgttagaccgtaaagggcttctctcacccgaaagtggtatcattcagccagctttatcattcttataatccagcatcatcttaaggacattttaataaacttgtttctttttttttttttttatgttggaaagacttaaggatcagaaggcaggaatgaggtgatgatagggatttgggtgggagagtgacacttaatcagagaaaatctgaaatgtgccttccagattcaaacacatcttgggacacgagcaggagtatctgcagcccgaatgggacttaaatttttgttctgttttattaagaagaagggaagtgttcttggcattagtaattattttttatctctcatcaattcaacctagaattttggattaatcacatctttattttattttatttttgtgttttctatggaggttcccaggctaggggtctaattggagctgtagccactggcccatgccacaatcacagcaacacgggatccgagacgagtctgcaatctacactacagctcacggcaacaccgaatccttaacccactgagcaaggccagggatcgaacccacaacctcatggtacctagtcagactcgttaaccactgagccacgagggaactcccctaatcacatctttaaattgtattatctgtacctgtaatctaaggccatctgccattgagaggaaaggagaaatcaggttgaatggagagaaaactcattctacacaaactgtaatttcctagtcatttggactgtcaggaaacttgttttaatttgaattaccttaaaaagggatttttgggtttttaaaggcttatccacgatgtaattgcccaattctccactttatcctatgctgttgagacacagtaagggttcttcccaccaggctgttgctccatgactggtgaggaagcttttgctggttccttgcagggtggctagttactcgatgtagatgaatcagatatgtatttattccattttgggaatacttggatcagtgtaattcagagatttactgctcatctgatatactgtcctatgtgggggcatctttttaaagccacaagttgctgcagttctgagaccacttatgtgacaagaggagacctgctcccacattgaggtctggcacagcttgtgtttctctttgccaaaaagggcaaaggccttgagcaagaagccagcattttcctaattacaaaactgaccacaattcctcgccaacctatcagtataaatcctttttttttttttctggtaatgcattattcctgtcagggagaactccacatttttcattatgatctggaatttatgcttaaacattaaccaatgttcaggcatcagctgatgcagtattgctgcatatgctataattggataaacttcataattaaatttttgaaagcagactctgatatgtaaaatctaaactgtatcaagccctctctatgtccagtctctcaactctttaattgtatggcatttagatagctgtcagtatcattttttacatactttacaaagtagtagaataaataccttagaacagaatatttcagtagctgatagcaaattttaaactatgaacacatgcttgaattcagaagtcttcttcagatatcagataatgggcagacagaaaaaaaaaaaaactgtgtgcaagttggtttatccggtatttcttttttaaagaaatttttaaattatagttgatttacaatgttgttccagtttctgctgtacagcaaagtgacccagtcatacacctatatacattcttatattatcttccatcatgttctatcctaagaaattggatatagttgctgtacaacaggacctcattgcttatccatttaaatgtaatagtttgcatcttaaccccaaactctcagtccatcccactccctttgtctatttcataactcagggatgtaaacttttcaattaagaagcaattgtgagttcctgctgtggctcggtgggttaagaacccaactagtatccatgaggatccgggttcaatccttggtctcacttagtaggttaaggatccagcattgctgtggctgtggtgtaggctggcagctgtagctctgattcgacccctatccttagaacttccatatgctgcaggtacagccctgaaaagacacaaaaaagaagcaagtggatgttgagcagtctctttgagagttctgaacactgcactgcttgtgcccctcacaagacactgaacatgtgggtcactggcgagacagtgtggcaatgtattccttgtaatgtaccaagtcttgccaaagcagtgaacaatattatgacacaacgttttgtcacagctggcccctaacaggagtgtgccagccagttcagccctggtcctgtgggagttgattcccacctctccagtatttggaaactgatgtcctgactcattggtgccttcaccagttctacaaccaggaatcttttccctttaatggattggccttttgcaagaaatagacaaaatatcagtgtgaatgacagcaaaccCCTATTCCATGCTGTCATGGGTGAAACTCTGGGAGATTCTCTTATTGACCCAGAAAGCGATGCCTTCGACACGCTGTCTGCAAACATTTCACAAG

Supplementary Figure 5

Putative C/EBPα and GATA-2 binding sites in the porcine *PPARγ2* promoter region

TFSEARCH searching transcription factor binding sites (ver. 1.3) was used to identify C/EBPα and GATA-2 binding sites in the 5-kb upstream region of the transcription initiation site of the porcine *PPARγ2* gene. The putative C/EBPα and GATA-2 binding sites are indicated by yellow and blue highlighting, respectively. The first exon of porcine *PPARγ2* is capitalized and the coding sequence is underlined. The wavy line shows GATA-2 sites overlapping. In total, 16 GATA-2 and 9 C/EBPα sites were identified.
